# Identification and evaluation of reference genes for quantitative real-time PCR analysis in *Polygonum cuspidatum* based on transcriptome data

**DOI:** 10.1186/s12870-019-2108-0

**Published:** 2019-11-14

**Authors:** Xiaowei Wang, Zhijun Wu, Wenqi Bao, Hongyan Hu, Mo Chen, Tuanyao Chai, Hong Wang

**Affiliations:** 1 College of Life Sciences, University of Chinese Academy of Sciences, Beijing, 100049 China; 2 Institute of Genetics and Developmental Biology, Chinese Academy of Sciences, Beijing, 100101 China

**Keywords:** *Polygonum cuspidatum*, Reference gene, RT-qPCR, Transcriptome, Phenylpropanoid pathway

## Abstract

**Background:**

*Polygonum cuspidatum* of the Polygonaceae family is a traditional medicinal plant with many bioactive compounds that play important roles in human health and stress responses. Research has attempted to identify biosynthesis genes and metabolic pathways in this species, and quantitative real-time PCR (RT-qPCR) has commonly been used to detect gene expression because of its speed, sensitivity, and specificity. However, no *P. cuspidatum* reference genes have been identified, which hinders gene expression studies. Here, we aimed to identify suitable reference genes for accurate and reliable normalization of *P. cuspidatum* RT-qPCR data.

**Results:**

Twelve candidate reference genes, including nine common (*ACT*, *TUA*, *TUB*, *GAPDH*, *EF-1γ*, *UBQ*, *UBC*, *60SrRNA*, and *eIF6A*) and three novel (*SKD1*, *YLS8*, and *NDUFA13*), were analyzed in different tissues (root, stem, and leaf) without treatment and in leaves under abiotic stresses (salt, ultraviolet [UV], cold, heat, and drought) and hormone stimuli (abscisic acid [ABA], ethylene [ETH], gibberellin [GA_3_], methyl jasmonate [MeJA], and salicylic acid [SA]). Expression stability in 65 samples was calculated using the △CT method, geNorm, NormFinder, BestKeeper, and RefFinder. Two reference genes (*NDUFA13* and *EF-1γ*) were sufficient to normalize gene expression across all sample sets. They were also the two most stable genes for abiotic stresses and different tissues, whereas *NDUFA13* and *SKD1* were the top two choices for hormone stimuli. Considering individual experimental sets, *GAPDH* was the top-ranked gene under ABA, ETH, and GA_3_ treatments, while *60SrRNA* showed good stability under MeJA and cold treatments. *ACT*, *UBC*, and *TUB* were suitable genes for drought, UV, and ABA treatments, respectively. *TUA* was not suitable because of its considerable variation in expression under different conditions. The expression patterns of *PcPAL, PcSTS*, and *PcMYB4* under UV and SA treatments and in different tissues normalized by stable and unstable reference genes demonstrated the suitability of the optimal reference genes.

**Conclusions:**

We propose *NDUFA13* and *EF-1γ* as reference genes to normalize *P. cuspidatum* expression data. To our knowledge, this is the first systematic study of reference genes in *P. cuspidatum* which could help advance molecular biology research in *P. cuspidatum* and allied species.

## Background

*Polygonum cuspidatum* is a perennial herb of the Polygonaceae family that is a well-known traditional Chinese medicine. Its first medicinal application records date back over 1800 years in *Mingyi Bielu* [1]. It has long been used in Chinese folk medicine for the treatment of abdominal masses, postpartum blood stasis, urethritis, suppuration, ulcers, sore throats, toothache, chronic bronchitis, hemorrhoids, and other ailments [1, 2]. More recently, oral intake or external applications of its processed products were shown to be effective in hepatitis, hypertension, hyperlipidemia, diabetes, jaundice, arthritis, skin burns, and scalds [3].

Numerous active compounds have been isolated and identified from *P. cuspidatum* to date, such as anthraquinones, flavonoids, stilbenes, organic acids, coumarins, catechins, and lignans [3]. The content of resveratrol, a stilbene, is higher in *P. cuspidatum* than in other plants [4], and several physiological and pharmacological studies have reported anti-oxidative, anti-cancer, anti-inflammatory, anti-tumor, anti-depressant, and anti-viral roles for resveratrol, as well as neuroprotective and metabolic regulation through interactions with multiple targets [5]. Other components of *P. cuspidatum* also show significant health-promoting effects in cells or animal models [6, 7]. Interestingly, the vast majority of active compounds are secondary metabolites synthesized during normal plant growth or in response to environmental stresses [8, 9]. However, the mechanism of *P. cuspidatum* responses to abiotic stresses and hormone stimuli as well as in various tissues needs to be thoroughly explored to better understand the production of these active ingredients.

Stilbenes and flavonoids, derivatives of the phenylpropanoid pathway, actively participate in the regulation of resistance to stresses, including pathogens, ultraviolet (UV) radiation, low/high temperature, drought, heavy metals, methyl jasmonate (MeJA), and ethylene (ETH) [8, 9]. Phenylalanine ammonia lyase (PAL) catalyzes the conversion of L-phenylalanine into cinnamic acid, which is the first committed step of the phenylpropanoid pathway. The same substrates 4-coumaroyl-CoA and malonyl-CoA are used by stilbene synthase (STS) and chalcone synthase to produce resveratrol and tetrahydroxychalcone, respectively, which are branch sites in the stilbene and flavonoid pathway [10]. Various transcription factors are involved in regulation of the phenylpropanoid pathway [11], and *MYB4* in *Arabidopsis* responds to UV and SA stresses to suppress phenylpropanoid pathway gene expression [12, 13].

The analysis of gene expression patterns can provide insights into complex metabolic processes. Quantitative real-time PCR (RT-qPCR) is commonly used to detect gene expression in different species because of its simplicity, speed, sensitivity, specificity, and high throughput [14]. However, despite these advantages, variability in initial materials, RNA integrity, RT-PCR efficiency, qPCR efficiency, and inherent technical variations will inevitably lead to errors, so it is necessary to use reference genes as internal controls [15]. An appropriate reference gene should be constantly expressed across the samples being investigated [15]. Classic reference genes for RT-qPCR data normalization, such as actin, tubulin, ubiquitin, elongation factor, translation initiation factor, and ribosomal RNA, are usually required for basic and essential processes in the cell. However, increasing experimental evidence shows that some of these genes are not as stable as previously thought [16]. Therefore, a host of highly stable novel reference genes have been identified using screening from genomes and transcriptome datasets [17, 18]. Previously, reference genes have not always been stable in different species and tissues, or at different developmental stages and experimental conditions within a single species [19]. Therefore, it is crucial to assess expression stability probabilities under specific conditions prior to use. Furthermore, two or more reference genes are desirable to avoid a biased or mistaken interpretation of the changes of target gene expression [20].

In this context, the △CT method [21], geNorm [22], NormFinder [23], BestKeeper [24], and RefFinder [25] have been developed to evaluate the stability of genes in biological samples. They have been successfully employed to validate reference genes in various medicinal plants, such as *Gentiana macrophylla* [26], *Achyranthes bidentata* [27], and *Euscaphis konishi*i [18]. In *P. cuspidatum*, *PcPKS1* [28], *PcPKS2* [29], *PcCHS1* [30], *PcSTS* [31], *PcMYB1* [32], and *PcWRKY33* [33] have been cloned and studied. However, the lack of *P. cuspidatum* reference genes has become a major hurdle for gene expression studies in this species. Because of a lack of sequence and expression information, we performed transcriptome sequencing of *P. cuspidatum* vegetative tissues (root, stem, and leaf) and leaves under different treatments (UV and MeJA) in our laboratory (unpublished data), providing a wealth of resources for our current selection of suitable reference genes.

The present study was undertaken to characterize candidate reference genes for RT-qPCR data normalization in *P. cuspidatum*. Twelve genes (*actin* [*ACT*], *tubulin-alpha* [*TUA*], *tubulin-beta* [*TUB*], *glyceraldehyde-3 phosphate dehydrogenase* [*GAPDH*], *elongation factor 1-gamma* [*EF-1γ*], *ubiquitin domain-containing protein* [*UBQ*], *ubiquitin-conjugating enzyme* [*UBC*], *60S ribosomal RNA* [*60SrRNA*], *eukaryotic translation initiation factor 6A* [*eIF6A*], *suppressor of K+ transport growth defect1* [*SKD1*], *thioredoxin-like protein* [*YLS8*], and *NADH dehydrogenase* [*ubiquinone*] *1 alpha subcomplex subunit 13-A* [*NDUFA13*]) were screened out from the *P. cuspidatum* transcriptome, and their expression levels were detected by RT-qPCR across all samples. These included three tissues (root, stem, and leaf), 32 abiotic-treated samples (salt, UV, cold, heat, and drought), and 30 hormone-treated samples (abscisic acid [ABA], ETH, gibberellin [GA_3_], MeJA, and salicylic acid [SA]). Expression stability was calculated using △CT, geNorm, NormFinder, BestKeeper, and RefFinder. Additionally, the expression of three target genes (*PcPAL*, *PcSTS*, and *PcMYB4*) under UV and SA treatments and in different tissues was normalized separately by the most and least stable genes to demonstrate the suitability of the recommended reference genes. Our results provide some useful resources for the future quantification of gene expression in *P. cuspidatum* and allied species.

## Results

### Identification of candidate reference genes

In this work, nine classic reference genes (*ACT*, *TUA*, *TUB*, *GAPDH*, *EF-1γ*, *UBQ*, *UBC*, *60SrRNA*, and *eIF6A*) and three novel reference genes (*SKD1*, *YLS8*, and *NDUFA13*) were identified from the *P. cuspidatum* transcriptome as candidate reference genes. The full-length cDNAs of these 12 genes, which were used to design specific qPCR primers, were submitted to NCBI GenBank (Table 1).

**Table 1:** Details of candidate reference genes and target genes used for RT-qPCR in *Polygonum cuspidatum*

Gene	Gene description	Primer sequence (5′-3′) Forward/Reverse	Product (bp)	*E* (%)	*R*^*2*^	NR accession	Arabidopsis Ortholog
*ACT*	actin 7	F:GCCGTCTATGATTGGAATGG R:TACCGTACAAGTCCTTCCTAA	99	98	0.999	MK288156	AT5G09810
*TUA*	Tubulin-alpha 6	F:CCAGATGCCAAGTGACAAAAC R:TTGTCTGTATGTTCCAGTCCT	154	86	0.998	MK288157	AT4G14960
*TUB*	Tubulin-beta 2	F:ATCCGACACTGTTGTTGAGC R:CCAAAGGATGGGGTTGAAAG	144	86	0.998	MK288158	AT5G23860
*GAPDH*	Glyceraldehyde-3 phosphate dehydrogenase	F:CAGTGACTGTTTTCGGTTGC R:AGCCTTGTCCTTGTCGGTGA	107	91	0.999	MK288159	AT1G13440
*EF-1γ*	Elongation factor 1-gamma	F:GTCATCCCTGATTGATTACGC R:GTGGGCAATAAAGCCAAGAC	112	95	0.998	MK288160	AT1G57720
*UBQ*	Ubiquitin domain-containing protein	F:AGTCCTCAACTTCGTGCTATG R:TTCTGGAGGCACATTTGGAGT	294	89	0.995	MK288161	AT2G17200
*UBC*	Ubiquitin-conjugating enzyme	F:ATTTGATGGCGTGGAGTTGC R:AGGGGTAAACATTGGGGTGG	156	98	0.999	MK288162	AT3G57870
*60SrRNA*	60S ribosomal RNA	F:ACTGTGATTTCGCAGACGCA R:CCTGGTGCTTGGTGAGACGG	124	98	0.999	MK288163	AT5G02610
*eIF6A*	Eukaryotic translation initiation factor 6A	F:CGGATCTTGACAGGGAAACC R:AACGGCACCTGAAGGAGTGT	187	94	0.997	MK288164	AT3G55620
*SKD1*	Suppressor of K+ transport growth defect1	F:GGCGATGGTGAGGGAGATGA R:ACCCAGCCACATCATTCCAC	106	104	0.997	MK288165	AT2G27600
*YLS8*	Thioredoxin-like protein YLS8	F:AGATCAACTGGGCTCTAAAGG R:AATCACCAGACCTCGACCCT	92	103	0.998	MK288166	AT5G08290
*NDUFA13*	NADH dehydrogenase [ubiquinone] 1 alpha subcomplex subunit 13-A	F:ATGTACCAGGTCGGCGTAGG R:TCCTTCATAATTCTGGCTTCC	161	98	0.998	MK288167	AT1G04630
Target gene							
*PcMYB4*	Transcription repressor MYB4	F:TTGATACCGCTACAACCACA R:CACCCGTCACAACGCTATT	196	96	0.994	MK288154	AT4G38620
*PcPAL*	Phenylalanine ammonia-lyase	F:AGAACAGGATCAAGGAAT R:AACAAGGAATCAATCATC	158	96	0.997	MK288155	AT2G37040
*PcSTS*	Stilbene synthases	F:CCAGACCTAACAGTTGAGA R:TCGCACCATCAGATTCA	80	90	0.997	EU647245	–

### Evaluation of primer specificity and efficiency

PCR products amplifying *P. cuspidatum* leaf cDNA were checked by 1% (m/v) agarose gel electrophoresis, which revealed a single band of expected size for each primer pair (Additional file 1: Figure S1). Additionally, a single peak in the melting curve for each gene confirmed primer specificity (Additional file 1: Figure S2). The qPCR efficiency (*E*) varied from 86% (*TUB*) to 103% (*SKD1*) with correlation coefficients (*R*^*2*^) ranging from 0.994 to 0.999 (Additional file 1: Figure S3). Hence each primer pair was highly efficient and specific to the targeted region.

### Expression profiling of candidate reference genes

**Fig. 1:**
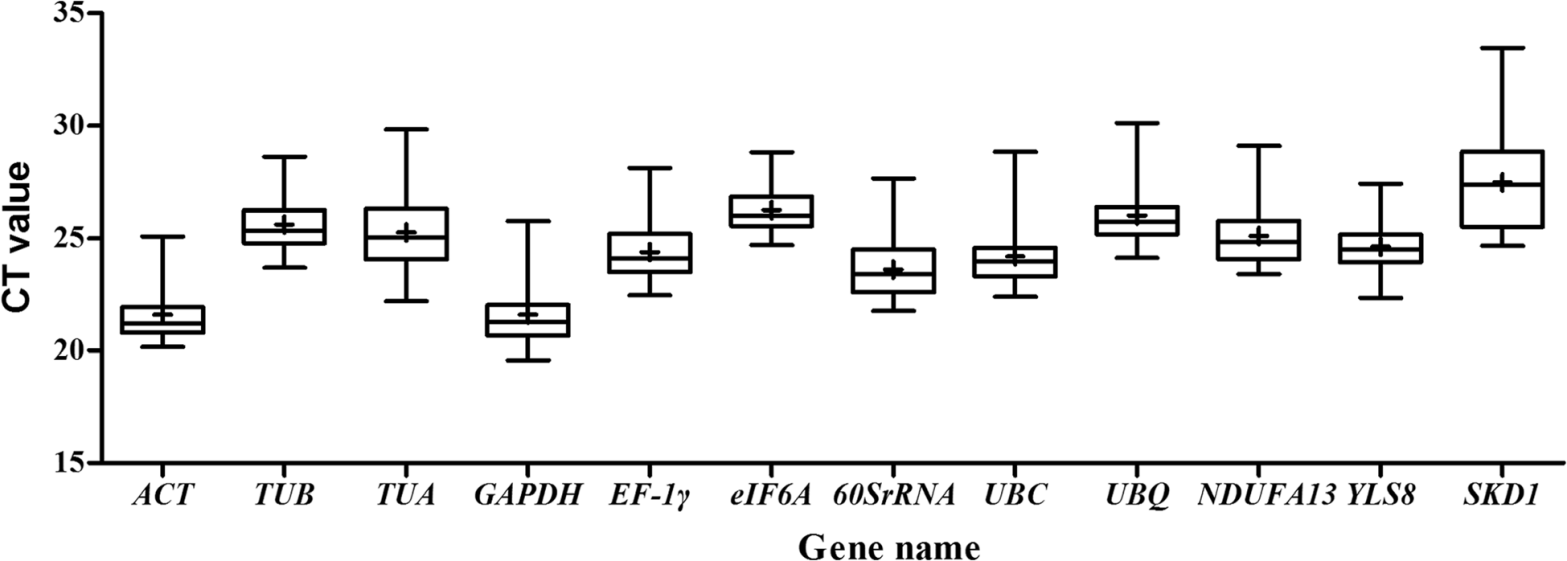
Boxplot analysis of cycle threshold (CT) values of 12 candidate reference genes across all samples. The boxes represent the interquartile range. The line across the box represents the median. The plus sign in the box show the mean values. Hyphens over and under the boxes is shown as the maximum and minimum, respectively

The expression levels of the 12 candidate reference genes (*ACT*, *TUA*, *TUB*, *GAPDH*, *EF-1γ*, *UBQ*, *UBC*, *60SrRNA*, *eIF6A*, *SKD1*, *YLS8*, and *NDUFA13*) were evaluated in 65 samples collected from different tissues without treatments and leaves under abiotic and hormone stimuli using CT values. Raw CT values of the 12 genes across all samples were shown in Additional file 2, and were plotted directly by Boxplot (Fig. 1). The CT values exhibited a relatively wide range from 19.84 (*GAPDH*) to 33.46 (*SKD1*) across all samples. Because the gene expression level is negatively correlated with the CT value, *ACT* was the highest expressed gene with the lowest mean CT value (21.60), whereas *SKD1* was the least abundant gene with the highest mean CT value (27.42) among the 12 genes. The expression variation among the 65 samples for each gene ranged from 4.12 (*eIF6A*) to 8.779 (*SKD1*). No gene showed an unchanged expression level under all conditions, so it was necessary to identify reference genes under specific experimental conditions in *P. cuspidatum.*

### Expression stability of candidate reference genes

In our study, each reference gene was evaluated in 11 experimental sets which were analyzed individually at first. To obtain a more comprehensive analysis, the sets were then divided into four different groups: (1) “Abiotic stress” (salt, UV, cold, heat, and drought) (2) “Hormone stimuli” (ABA, ETH, GA_3_, MeJA, and SA) (3) “Different tissues” (root, stem, and leaf), and (4) “All” (all experimental sets). More specifically, the stability of the 12 candidate reference genes was analyzed by the △CT method, geNorm, NormFinder, BestKeeper, and RefFinder.

**Fig. 2:**
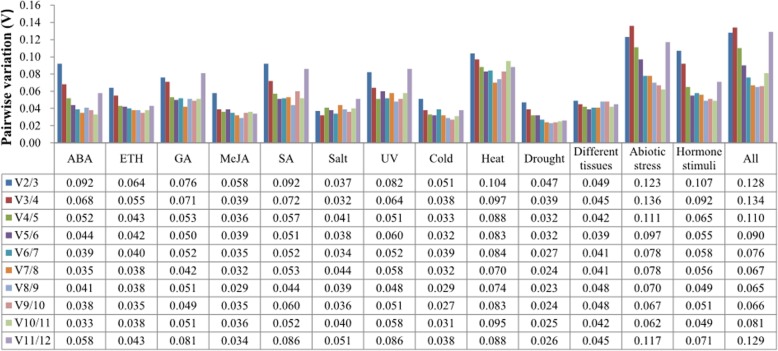
Determination of the optimal numbers of reference genes for normalization in *Polygonum cuspidatum*

geNorm estimates the optimal number of reference genes required for accurate and reliable RT-qPCR normalization. As shown in Fig. 2, V_2/3_ values were much lower than the cut-off value of 0.15 for all different groups, indicating that two reference genes would be sufficient for accurate and reliable normalization of the gene expression data.

For “Abiotic stress”, *60SrRNA* and *EF-1γ* had the lowest *M* value (0.25) followed by *NDUFA13* (0.35), indicating that these were the three most stable genes in geNorm analysis. *NDUFA13* and *EF-1γ* were the two top-ranking genes in NormFinder and △CT analysis, while *eIF6A* and *60SrRNA* were top in BestKeeper analysis. According to the calculations performed by RefFinder, *EF-1γ* and *NDUFA13* were the most appropriate reference genes under abiotic stress conditions. The comprehensive ranking order for every specific abiotic stress was not entirely consistent. *EF-1γ*, *NDUFA13*, and *60SrRNA* were also identified as the three most stable reference genes under heat treatment, but *EF-1γ* and *NDUFA13* fell outside the list of the four most stable genes, though *60SrRNA* ranked top under cold conditions. For salt treatment, *YLS8* was the top-ranked gene, followed by *EF-1γ*, *TUB*, and *NDUFA13*. For UV treatment, *UBC* had the highest stability, while *EF-1γ*, *NDUFA13*, and *60SrRNA* also exhibited good stability. *NDUFA13* was the optimal reference gene under drought treatment, followed by *EF-1γ* and *60SrRNA* with low *CVs* (Table 2, Additional file 3: Table S1).

**Table 2:** Gene expression stability under multiple conditions ranked by △CT, BestKeeper, NormFinder, geNorm and RefFinder

Groups	Ranking	RefFinder		△CT		BestKeeper		Normfinder		geNorm	
		Gene	Stability	Gene	Stability	Gene	Stability	Gene	Stability	Genes	Stability
Abiotic stress	1	*EF-1γ*	1.86	*NDUFA13*	0.64	*eIF6A*	0.89	*NDUFA13*	0.17	*60SrRNA*	0.28
	2	*NDUFA13*	1.97	*EF-1γ*	0.66	*60SrRNA*	0.93	*EF-1γ*	0.22	*EF-1γ*	0.28
	3	*60SrRNA*	2.06	*60SrRNA*	0.70	*EF-1γ*	0.96	*60SrRNA*	0.33	*NDUFA13*	0.35
	4	*eIF6A*	4.30	*ACT*	0.75	*YLS8*	0.97	*UBQ*	0.44	*UBQ*	0.47
	5	*UBQ*	4.68	*UBQ*	0.76	*NDUFA13*	1.01	*ACT*	0.47	*ACT*	0.53
	6	*ACT*	5.32	*TUB*	0.77	*TUB*	1.05	*TUB*	0.52	*TUB*	0.58
	7	*TUB*	6.24	*eIF6A*	0.79	*UBQ*	1.05	*eIF6A*	0.54	*eIF6A*	0.60
	8	*YLS8*	7.52	*UBC*	0.83	*ACT*	1.07	*UBC*	0.57	*YLS8*	0.63
	9	*UBC*	9.16	*TUA*	0.86	*TUA*	1.07	*TUA*	0.64	*GAPDH*	0.66
	10	*TUA*	9.24	*YLS8*	0.89	*UBC*	1.09	*YLS8*	0.72	*TUA*	0.69
	11	*GAPDH*	10.46	*GAPDH*	0.89	*GAPDH*	1.22	*GAPDH*	0.72	*UBC*	0.71
	12	*SKD1*	12.00	*SKD1*	1.46	*SKD1*	1.41	*SKD1*	1.39	*SKD1*	0.83
Hormone stimuli	1	*NDUFA13*	1.97	*NDUFA13*	0.49	*ACT*	0.33	*NDUFA13*	0.26	*60SrRNA*	0.34
	2	*SKD1*	2.63	*SKD1*	0.50	*SKD1*	0.36	*SKD1*	0.26	*EF-1γ*	0.34
	3	*eIF6A*	3.41	*eIF6A*	0.50	*NDUFA13*	0.43	*eIF6A*	0.28	*eIF6A*	0.36
	4	*EF-1γ*	3.98	*YLS8*	0.51	*UBC*	0.44	*YLS8*	0.31	*YLS8*	0.39
	5	*YLS8*	4.43	*EF-1γ*	0.53	*eIF6A*	0.44	*EF-1γ*	0.33	*NDUFA13*	0.39
	6	*60SrRNA*	4.46	*60SrRNA*	0.53	*YLS8*	0.45	*60SrRNA*	0.34	*SKD1*	0.40
	7	*ACT*	5.62	*UBQ*	0.56	*UBQ*	0.47	*UBQ*	0.39	*UBC*	0.42
	8	*UBC*	6.51	*UBC*	0.58	*TUB*	0.52	*UBC*	0.42	*UBQ*	0.45
	9	*UBQ*	7.24	*GAPDH*	0.59	*EF-1γ*	0.52	*GAPDH*	0.43	*GAPDH*	0.46
	10	*GAPDH*	8.74	*ACT*	0.62	*GAPDH*	0.52	*ACT*	0.47	*ACT*	0.49
	11	*TUB*	10.46	*TUB*	0.66	*60SrRNA*	0.56	*TUB*	0.51	*TUB*	0.52
	12	*TUA*	12.00	*TUA*	0.92	*TUA*	0.79	*TUA*	0.84	*TUA*	0.58
Different tissues	1	*NDUFA13*	1.57	*NDUFA13*	0.31	*GAPDH*	0.92	*ACT*	0.06	*NDUFA13*	0.10
	2	*EF-1γ*	2.45	*EF-1γ*	0.32	*NDUFA13*	1.03	*NDUFA13*	0.10	*EF-1γ*	0.10
	3	*ACT*	2.63	*ACT*	0.32	*EF-1γ*	1.07	*60SrRNA*	0.10	*60SrRNA*	0.13
	4	*60SrRNA*	3.13	*60SrRNA*	0.32	*60SrRNA*	1.08	*EF-1γ*	0.13	*ACT*	0.16
	5	*GAPDH*	4.43	*eIF6A*	0.37	*TUB*	1.11	*eIF6A*	0.22	*TUB*	0.19
	6	*TUB*	5.69	*TUB*	0.38	*ACT*	1.19	*UBQ*	0.26	*GAPDH*	0.21
	7	*eIF6A*	6.32	*UBQ*	0.39	*TUA*	1.23	*TUB*	0.27	*UBQ*	0.24
	8	*UBQ*	7.17	*GAPDH*	0.42	*eIF6*	1.24	*GAPDH*	0.32	*eIF6A*	0.27
	9	*YLS8*	9.24	*YLS8*	0.46	*UBQ*	1.30	*YLS8*	0.38	*YLS8*	0.31
	10	*TUA*	10.49	*UBC*	0.54	*YLS8*	1.34	*SKD1*	0.47	*UBC*	0.35
	11	*UBC*	10.49	*SKD1*	0.54	*UBC*	1.40	*UBC*	0.50	*SKD1*	0.38
	12	*SKD1*	10.98	*TUA*	0.57	*SKD1*	1.46	*TUA*	0.54	*TUA*	0.41
All	1	*NDUFA13*	2.28	*NDUFA13*	0.68	*YLS8*	0.78	*NDUFA13*	0.21	*60SrRNA*	0.31
	2	*EF-1γ*	2.30	*EF-1γ*	0.68	*eIF6A*	0.84	*EF-1γ*	0.24	*EF-1γ*	0.31
	3	*60SrRNA*	3.08	*60SrRNA*	0.71	*ACT*	0.90	*60SrRNA*	0.31	*NDUFA13*	0.38
	4	*UBQ*	4.00	*UBQ*	0.75	*UBQ*	0.91	*UBQ*	0.42	*UBQ*	0.48
	5	*eIF6A*	4.36	*ACT*	0.77	*UBC*	0.93	*ACT*	0.46	*eIF6A*	0.54
	6	*ACT*	4.79	*eIF6A*	0.78	*TUB*	1.00	*eIF6A*	0.50	*TUB*	0.57
	7	*YLS8*	5.62	*TUB*	0.80	*EF-1γ*	1.01	*TUB*	0.50	*ACT*	0.59
	8	*TUB*	6.48	*UBC*	0.83	*GAPDH*	1.02	*UBC*	0.53	*GAPDH*	0.61
	9	*UBC*	7.33	*GAPDH*	0.83	*60SrRNA*	1.04	*GAPDH*	0.58	*UBC*	0.63
	10	*GAPDH*	8.49	*YLS8*	0.94	*NDUFA13*	1.04	*YLS8*	0.77	*YLS8*	0.66
	11	*TUA*	11.00	*TUA*	1.05	*TUA*	1.29	*TUA*	0.85	*TUA*	0.72
	12	*SKD1*	12.00	*SKD1*	1.62	*SKD1*	1.93	*SKD1*	1.54	*SKD1*	0.87

For “Hormone stimuli”, the best reference genes were *60SrRNA* and *EF-1γ* in geNorm analysis. *NDUFA13* and *SKD1* were the two most stable reference genes by both △CT and NormFinder, but *ACT* and *SKD1* were identified by BestKeeper. In a comprehensive analysis, *NDUFA13* and *SKD1* were the two optimal reference genes under hormone stimuli conditions. When considering every hormone treatment, *NDUFA13* was one of the three most stable reference genes for all hormone treatments except ABA; *GAPDH* and *SKD1* were among the four most stable genes under ABA, ETH, and GA conditions (Table 2, Additional file 3: Table S1).

For “Different tissues”, *NDUFA13* and *EF-1γ* were the most stable combination in geNorm analysis. Similar results were seen by △CT. *NDUFA13* and *EF-1γ* next to *GAPDH* were the top-ranking genes in NormFinder analysis. In BestKeeper analysis, *ACT* ranked first with the lowest stability value (0.06), while *NDUFA13* and *EF-1γ* ranked second and fourth, respectively. *NDUFA13* and *EF-1γ* were recommended in different tissues of *P. cuspidatum* by RefFinder (Table 2, Additional file 3: Table S1).

When all samples were taken into account, *60SrRNA*, *EF-1γ*, and *NDUFA13* ranked most highly in geNorm, △CT, and NormFinder analysis. However, they were low in the ranking in BestKeeper analysis. RefFinder ranked the candidate reference genes from the highest to the lowest stability as follows: *NDUFA13* > *EF-1γ* > *60SrRNA* > *UBQ* > *eIF6A* > *ACT* > *YLS8* > *TUB* > *UBC* > *GAPDH* > *TUA* > *SKD1* (Table 2). Taken together, *NDUFA13* and *EF-1γ* were the two most suitable reference genes across all samples of *P. cuspidatum*. Additionally, it was clear that *TUA* was an unstable gene under all experiment conditions according to all valuation systems (Table 2, Additional file 3: Table S1).

### Validation of candidate reference genes

To ensure the accuracy and reliability of our results, the relative expression patterns of *PcPAL*, *PcSTS*, and *PcMYB4* were analyzed under Abiotic stress (UV), Hormone stimuli (SA), and in different tissues (leaf, stem, and root). The two most stable reference genes (*NDUFA13* and *UBQ* for SA, *UBC* and *EF-1γ* for UV, *NDUFA13* and *EF-1γ* for different tissues) and one unstable gene (*TUA*) were selected for normalizing qPCR data.

**Fig. 3:**
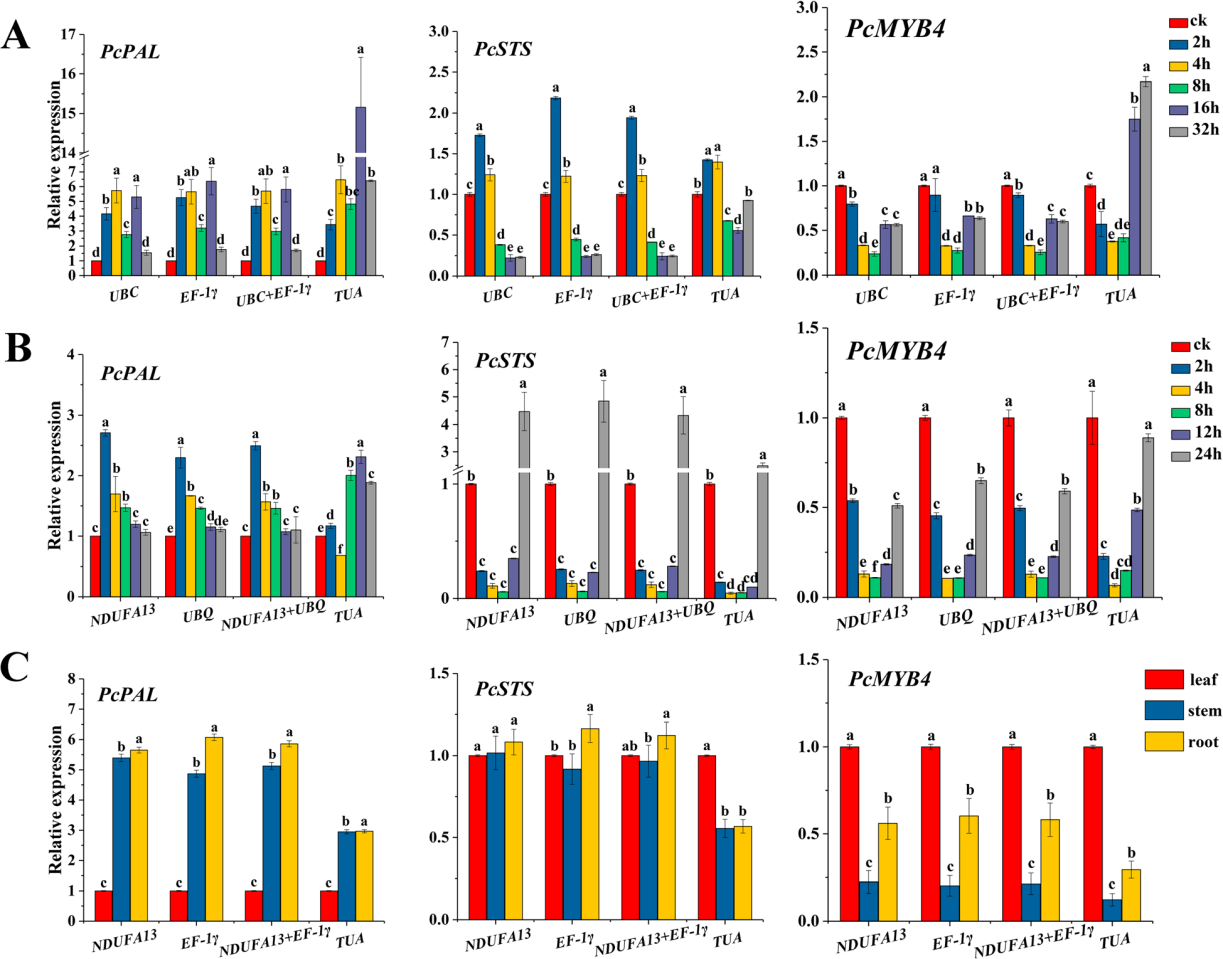
Relative expression patterns of target genes for UV (**A**), SA (**B**) and different tissues (**C**). The most stable reference genes (*NDUFA13* and *UBQ* for SA, *UBC* and *EF-1γ* for UV, *NDUFA13* and *EF-1γ* for tissues) and unstable gene (*TUA*) were selected for normalizing qPCR data. Data are represented as mean ± *SD*, different letters on the vertical bars indicate significant difference at 0.05 levels

As shown in Fig. 3a, under UV treatment, *PcPAL* was significantly induced at all analyzed stress times. However, the expression levels at 16 h and 32 h with *TUA* normalization were much higher than those with either *UBC*, *EF-1γ*, or their geometric mean. The expression of *PcMYB4* was inhibited by UV treatment, initially reduced after reaching the lowest level at 8 h, then increased afterwards. However, the expression levels of *PcMYB4* at 16 h and 32 h were overestimated with *TUA* normalization. Similar misjudgments occurred in the analysis of *PcSTS* expression data. Some divergences in the results were also observed under SA treatment (Fig. 3b). The expression level of *PcPAL* with *NDUFA13*, *UBQ*, or their geometric mean achieved the highest after adding SA for 2 h and was higher at 4 h than the control, whereas the opposite results were displayed with *TUA* normalization. *PcSTS* and *PcMYB4* responded quickly to SA, and a low expression level was maintained for a long time (2–12 h). *TUA* normalization similarly led to erroneous interpretation of the relative expression patterns of *PcMYB4*. In different tissues (Fig. 3c), the expression patterns of *PcPAL*, *PcSTS*, and *PcMYB4* were similar when *NDUFA13*, *EF-1γ*, and *TUA* were used for normalization, but the fold-changes in root and stem were underestimated with normalization by *TUA*.

Overall, the expression patterns of *PcPAL*, *PcSTS*, or *PcMYB4* were nearly the same when using stable reference genes for normalization, whereas the expression levels had large variations when *TUA* was used. Furthermore, the RT-qPCR results normalized by the stable reference genes under UV treatment and in different tissues were more consistent with the target gene expression profiling derived from *P. cuspidatum* transcriptome data (Additional file 3: Table S2). Thus, the most suitable reference genes calculated by the above mentioned software were applicable under some specific conditions.

## Discussion

RT-qPCR is one of the most commonly used techniques to obtain gene expression profiles in molecular biology. A prerequisite of this is the selection of appropriate reference genes for data normalization to ensure the accuracy and reliability of the results [15]. Large-scale gene segments and gene expression data generated by sequencing provide abundant resources for the identification and evaluation of reference genes, especially in non-model species [18, 26].

In this work, we made full use of transcriptome sequencing data available in our laboratory to identify candidate reference genes for *P. cuspidatum*. The expression stability was evaluated by △CT method, geNorm, NormFinder, and BestKeeper, although the results obtained were not completely consistent. Such discrepancies in stability ranking were also reported in some previous studies [34]. Interestingly, we found that the rankings by △CT, geNorm, and NormFinder, were similar, especially for individual sets, but different from those by BestKeeper; similar findings were reported in other studies [19, 34]. For example, in our work, under ETH, GA_3_, and SA treatment, *ACT* was identified as the most stable gene by BestKeeper analysis, but performed unsatisfactorily in △CT, NormFinder, and geNorm analysis. Therefore, comprehensive analysis with RefFinder of multiple results from different software could help select a more appropriate reference gene.

*ACT*, *TUA*, and *TUB*, which encode cytoskeletal proteins, are extensively used as reference genes, and their high stability is reported in many plants [35, 36]. However, we showed that *TUA* performed poorly across all sample sets, *ACT* performed less well in most cases, and *TUB* was more affected by environmental factors such as SA and cold; this was similar to previous results obtained in *Eucommia ulmoides* [37]. *GAPDH* encodes an abundant glycolytic enzyme present in most cell types, and has universally been used as a reference gene in RT-qPCR. *GAPDH* showed a good performance in response to “Hormone stimuli” in parsley and carrot leaves, but was less stable for most abiotic stresses [36, 38]. The performance of *GAPDH* in our study was relatively consistent with those of previous studies, except for responses to MeJA and drought. Ubiquitin is a small regulatory protein found in most tissues of eukaryotic organisms. We showed that *UBC* was the most stable gene under UV treatment, in agreement with the findings of Borges [39]. Moreover, *UBQ* was one of the two most stable genes under SA treatment, which differed from the results obtained in *Achyranthes bidentata* [27] and carrot [36]. However, *UBC* and *UBQ* did not perform well across all other sets, which is consistent with the results seen in parsley [38]. Thus, *UBC* and *UBQ* were not the preferred gene choices in our study.

Eukaryotic translation initiation factor and ribosomal RNA have been reported as reference genes in many studies, such as *eIF4a* in *Eleusine indica* [40], and *eIF5A* and *60S ribosomal RNA* in *Panax ginseng* [41]. In our study, the performances of *eIF6A* and *60SrRNA* were not always the best in “Abiotic stress”, “Hormone stimuli”, and “all” groups, but were within an acceptable range. Elongation factor 1 (EF-1), composed of the four subunits EF-1α, EF-1β, EF-1δ, and EF-1γ, plays a central role in protein biosynthesis [42]. *EF-1α*, encoding a G-protein, was used for the normalization of qPCR data in some medicinal plants such as *G. macrophylla* [26] and *A. bidentata* [27]. The main function of EF-1γ is to ensure the correct scaffolding of different subunits in the EF-1 complex as well as to direct its intracellular localization [43]. The expression stability of *EF-1γ* had previously been evaluated in *P. ginseng* [34] and *Nilaparvata lugens* [44]. In our study, *EF-1γ* was one of the two most suitable reference genes with stable expression levels under various experimental conditions.

Novel reference genes such as *SKD1*, *YLS8*, and *NDUFA13* have also been selected for gene normalization by the application of large amounts of omics data. *SKD1*, encoding a protein that contributes to vacuolar trafficking and maintenance of the large central vacuole of plant cells [45], was first selected as a reference gene in pear [46]. In this study, *SKD1* was preferable under “Hormone stimuli” conditions, but had to be discarded under “Abiotic stress” and “Different tissues” conditions because of its low expression and high variation. *YLS8* encodes a protein involved in mitosis, and performed well under ABA, MeJA, and salt conditions, but poorly in other individual sets and the three combination groups. These results were not identical to those obtained previously [46, 47]. Notably, *NDUFA13* was remarkably stable in the individual sets (except in the ABA group) as well as in the combination groups, suggesting it is an almost ideal reference gene. NDUFA13, encoded by the nuclear genome, is an accessory subunit of the mitochondria respiratory chain complex I, which transfers electrons from nicotinamide adenine dinucleotide to ubiquinone [48]. The fact that mitochondria are known as eukaryotic cell powerhouses may explain why *NDUFA13* showed stable expression under various conditions in this study. *NDUFA13* was previously reported as a reference gene in *Apostichopus japonicus* [49], but no studies have investigated its potential in plants. In this regard, the present study is the first known report of *NDUFA13* as a reference gene in plants.

*PAL*, *STS*, and *MYB*4 are three important regulating genes in the phenylpropanoid pathway. When using the stable reference genes, the relative expression patterns of *PcPAL* and *PcMYB4* were in agreement with previous reports; for example, *MYB4* was down-regulated by exposure to UV-B light in *Arabidopsis thaliana* [12] and *Brassica rapa* [50], and *PAL* was induced by exposure to UV and SA in *A. thaliana* [51] and *Juglans regia* [52]. To our knowledge, *STS* is found in only a few plants, and not in *Arabidopsis* or tobacco. Research into *STS* expression is currently concentrated in grapes, where it was shown to be strongly induced by UV-C [53] and SA [54]. We observed the opposite expression patterns of *STS* in response to SA in *P. cuspidatum*, with rapidly decreasing expression lasting for 12 h. These differences may reflect variations in UV wavelengths or species. Moreover, our results are consistent with *P. cuspidatum* transcriptome data. Evidently, there were obvious underestimates or overestimates in our results following normalization by the least stable gene, *TUA*. Our results demonstrate the importance of using a stable reference gene for normalization to obtain accurate results.

Based on the above analysis, we suggest that no single gene should be used for normalization in all species, tissues, or treatments. Therefore, suitable reference genes for given species and conditions should be explored.

## Conclusions

We evaluated the expression stability of 12 candidate reference genes in different tissues of *P. cuspidatum* and under different treatment conditions. *NDUFA13* and *EF-1γ* were identified as the best two reference genes for normalizing RT-qPCR gene expression data. Their reliability and effectiveness were verified by *PcPAL*, *PcSTS*, and *PcMYB4*. To our knowledge, the current work is the first systematic analysis of suitable reference genes that will facilitate further research into the molecular biology of *P. cuspidatum* and other closely related species.

## Methods

### Plant material

Seeds of *P. cuspidatum* were collected from the medicinal plant garden of the Institute of Botany, Chinese Academy of Sciences (Beijing, China). Seeds were surface-sterilized and sown on Murashige and Skoog (MS) agar medium in a growth chamber at 24 °C with a 16 h/8 h light/dark cycle. After 1 month, similar seedlings were submitted to different treatments. For hormone treatment, the seedlings were uniformly sprayed with 0.5 mM MeJA, 1 mM SA, 0.1 mM ABA, 0.5 mM GA_3_ or 0.5 g/L ethrel. For cold and heat treatments, the seedlings were respectively kept in 4 °C and 42 °C illumination boxes. For UV irradiation, the seedlings were placed under a UV-B transilluminator for 20 min. For salt treatment, the seedlings were transplanted to MS agar medium containing 100 mM NaCl. Leaf samples were collected at 0, 2, 4, 8, 16, and 32 h after UV and MeJA treatments, and 0, 2, 4, 8, 12, and 24 h for the other treatments. For drought treatment, plants grown for 2 months in plastic pots containing a soil/vermiculite mixture (1:1) under the same conditions were kept in dry soil for 1 week, then rehydrated and sampled daily (0, 1, 2, 3, 4, 5, 6, 7, and 8 d). Tissue-specific samples (root, stem, and leaf) were collected from plants grown for 2 months in the soil. All 65 samples, including three tissue-specific samples (root, stem, and leaf), 32 stress-treated samples (salt-, UV-, cold-, heat-, and drought-treated leaves), and 30 hormone-treated samples (ABA-, ETH-, GA_3_-, MeJA-, and SA-treated leaves), were separately collected in three biological repeats. All samples were immediately frozen in liquid nitrogen and stored at − 70 °C before RNA extraction.

### RNA isolation and cDNA synthesis

Total RNA was isolated from samples using the Plant Total RNA Purification Kit (GeneMark, Taiwan, China) following the manufacturer’s instructions. The RNA integrity was checked on a 1% (m/v) agarose gel. The quantity and quality of the total RNA samples were assessed by recording absorbances at 260/280 nm and 260/230 nm with the NanoDrop 2000 spectrophotometer (Thermo Fisher Scientific, Waltham, MA, USA). Only RNA samples 1.8 < OD_260/280_ < 2.2 and OD_260/230_ > 2.0 were used for subsequent cDNA synthesis. Total RNA (3 μg) was reverse-transcribed into cDNA using the Hifair™ II 1st Strand cDNA Synthesis SuperMix (Yeasen, Shanghai, China) with oligo (dT) primers according to the manufacturer’s instructions. cDNA was diluted at 1:20 with the EASY dilution solution (Takara, Japan), then stored at − 20 °C until required as template for qPCR.

### Selection of candidate reference genes

The expression levels of unigenes are commonly estimated by fragments per kilobase of transcript per million mapped reads values [55]. A candidate reference gene should be moderately expressed with a small coefficient of variation (*CV*) [56]. In this work, the *CV* calculation formula was: *CV* = standard deviation of reads per kilobase of transcript per million mapped reads (RPKM)/average RPKM. We downloaded the sequences of nine classic reference genes (*ACT*, *TUA*, *TUB*, *GAPDH*, *EF-1γ*, *UBQ*, *UBC*, *60SrRNA*, and *eIF6A*) identified in other species, and carried out BLASTn queries against the *P. cuspidatum* transcriptome. Then, the highest ortholog sequences were checked against the *Arabidopsis* genome database and recorded in Table 1. Additionally, three new candidate genes, *SKD1*, *YLS8*, and *NDUFA13*, were selected based on their low *CV* and appropriate RPKM values (Additional file 3: Table S3). The cDNA and genomic DNA sequences of these candidate reference genes were shown in Additional file 4 and Additional file 5, respectively.

### Primer design

Specific primers were designed using Primer 5 software and synthesized by GENEWIZ Company (Tianjin, China). Gene characteristics and primer details are shown in Table 1. The specificity of each primer pair was assessed by amplification and melting curve analysis. The correlation coefficient (*R*^*2*^) and amplification efficiency (*E*) for all primer pairs were evaluated by standard curves using fourfold dilutions of the pooled cDNA (1/4, 1/16, 1/64, 1/256, and 1/1024).

### RT-qPCR

All qPCR was carried out in 96-well plates using QuantStudio™ Real-Time PCR Software (Applied Biosystems, USA) with the Hieff™ qPCR SYBR® Green Master Mix (Yeasen, Shanghai, China). Each 10 μL reaction included: 5.0 μL of 2× Hieff™ qPCR SYBR® Green Master Mix, 0.2 μL of each primer (10 μM), 1.0 μL of diluted (1:20) cDNA template, and 3.6 μL of RNase-free water. The cycle program for product amplification was: 95 °C for 5 min (hot-start activation) followed by 40 cycles of 95 °C for 10 s (denaturation), 58 °C for 20 s (annealing), and 72 °C for 20 s (extension). The melting curve was generated after 40 cycles to test the specificity of each primer pair across the temperature range of 60–95 °C at a heating rate of 0.05 °C/s. Three technical replicates were used for each sample.

### Data analysis and assessment of candidate reference genes performance

The CT values represent the expression level of each candidate reference gene. The amplification efficiency was calculated by: *E* (%) = (10 ^− 1/slope^ − 1) × 100 [57]. The stability of gene expression was evaluated by the △CT method [21], geNorm [22], NormFinder [23], BestKeeper [24], and RefFinder [25].

The △CT method was employed to rank the genes by calculating the average standard deviation (*SD*) based on the relative expression of all pairwise combinations of candidate reference genes. The gene with the lowest *SD* was identified as the most stable reference gene [21]. The geNorm calculates the expression stability value (*M*-value) for each gene. Genes with the lowest *M* values have the most stable expression. geNorm determines the pairwise variations (*V*) of one specific gene with all others. A cut-off value of V_n/n + 1_ < 0.15 means that further addition of reference genes no longer makes any significant contribution to the normalization. For instance, V_2/3_ < 0.15 means that two reference genes are sufficient for data normalization [22]. NormFinder provides a stability value (*SV*) for each gene, which takes intergroup and intragroup relationships into consideration. A lower SV indicates a higher stability [23]. BestKeeper calculates three variables based on the CT values of all genes: the coefficient of correlation (*r*), standard deviation (*SD*), and coefficient of variance (*CV*). A more stable gene exhibits a lower *SD* ± *CV* value [24]. RefFinder gives a comprehensive ranking for each candidate reference gene based on the geometric mean of the weights of all genes calculated by the above four computational approaches. A lower geometric mean of the ranking values indicates a more stable expression [25].

### Validation of selected candidate reference genes

To validate the selected reference genes, qPCR was performed to analyze target gene expression levels (*PcMYB4*, *PcPAL*, and *PcSTS*) under UV and SA treatments and in different tissues. The homologous *P. cuspidatum* genes *AtMYB4* and *AtPAL1* were cloned and named *PcMYB4* and *PcPAL*, respectively (Table 1, Additional file 4, Additional file 5). *PcSTS* (EU647245.1) was isolated in previous studies from our laboratory [31]. Primer design and detection for these three genes were performed according to the aforementioned methods. Relative expression levels were calculated with the 2 ^−△△CT^ method.

## Supplementary information

**Additional file 1: Figure S1.** PCR amplification patterns of the 12 candidate reference genes and 3 target genes. Bands were targeted to *ACT* (1), *TUA* (2), *TUB* (3), *GAPDH* (4), *EF-1γ* (5), *NDUFA13* (6), *UBQ* (7), *UBC* (8), *60SrRNA* (9), *SKD1* (10), *YLS8* (11), *eIF6A* (12), *PcMYB4* (13), *PcPAL* (14), *PcSTS* (15). **Figure S2.** Melting curves of 12 candidate reference genes and 3 target genes in *Polygonum cuspidatum.* **Figure S3.** Standard curves of 12 candidate reference genes and 3 target genes in *Polygonum cuspidatum*.

**Additional file 2: **Raw CT values of the 12 candidate reference genes across all samples of *Polygonum cuspidatum*.

**Additional file 3: Table S1**. Gene expression stability ranked by △CT, BestKeeper, NormFinder, geNorm and RefFinder under individual condition. **Table S2.** RPKM values of 3 target genes in *Polygonum cuspidatum* transcriptome. **Table S3.** RPKM values of 3 novel genes covering 7 transcriptomes data of *Polygonum cuspidatum*.

**Additional file 4.** cDNA sequences of 12 candidate reference genes and 3 target genes. Coding sequence (CDS) were marked green.

**Additional file 5.** Genomic DNA sequences of 12 candidate reference genes and 3 target genes. The exons were shown in green shading, qPCR primers were marked yellow.

## Data Availability

The data and materials supporting the conclusions of this study are included within the article and its additional files.

## Abbreviations

*60SrRNA*: 60S ribosomal RNA
ABA: Abscisic acid
*ACT*: Actin 7
*EF-1γ*: Elongation factor 1-gamma
*eIF6A*: Eukaryotic translation initiation factor 6A
ETH: Ethylene
GA3: Gibberellin
*GAPDH*: Glyceraldehyde-3 phosphate
MeJA: Methyl jasmonate
*MYB4*: Transcription repressor MYB4
*NDUFA13*: NADH dehydrogenase [ubiquinone] 1 alph
*PcPAL*: Phenylalanine ammonia-lyase
*PcSTS*: Stilbene synthases
SA: Salicylic acid
*SKD1*: Suppressor of K+ transport growth defect1
*TUA*: Tubulin-alpha 6
*TUB*: Tubulin-beta 2
*UBC*: Ubiquitin-conjugating enzyme
*UBQ*: Ubiquitin domain-containing
UV: Ultraviolet
*YLS8*: Thioredoxin-like protein YLS8
