# Supplementary material for: Identification and evaluation of reference genes for quantitative real-time PCR analysis in Polygonum cuspidatum based on transcriptome data
Source: BMC Plant Biol. 2019 Nov 14;19:498. doi: 10.1186/s12870-019-2108-0 (PMC6854638; doi:10.1186/s12870-019-2108-0)
Supplement: Supplementary file 1 — Additional file 1: Figure S1. PCR amplification patterns of the 12 candidate reference genes and 3 target genes. Bands were targeted to ACT (1), TUA (2), TUB (3), GAPDH (4), EF-1γ (5), NDUFA13 (6), UBQ (7), UBC (8), 60SrRNA (9), SKD1 (10), YLS8 (11), eIF6A (12), PcMYB4 (13), PcPAL (14), PcSTS (15). Figure S2. Melting curves of 12 candidate reference genes and 3 target genes in Polygonum cuspidatum. Figure S3. Standard curves of 12 candidate reference genes and 3 target genes in Polygonum cuspidatum. [file 12870_2019_2108_MOESM1_ESM.docx]

**
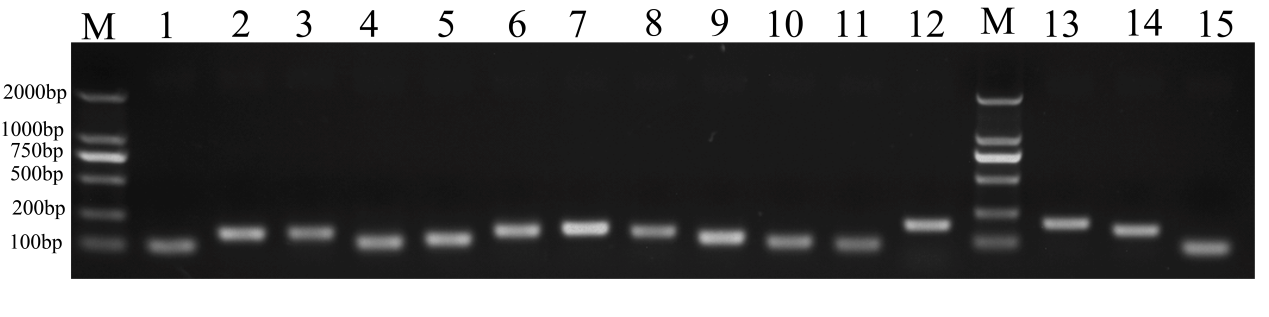
**

**Figure S1.** PCR amplification patterns of the 12 candidate reference genes and 3 target genes.

Bands were targeted to *ACT* (1), *TUA* (2), *TUB* (3), *GAPDH* (4), *EF-1γ* (5), *NDUFA13* (6), *UBQ* (7), *UBC* (8), *60SrRNA* (9), *SKD1* (10), *YLS8* (11), *eIF6A* (12), *PcMYB4* (13), *PcPAL* (14), *PcSTS* (15).


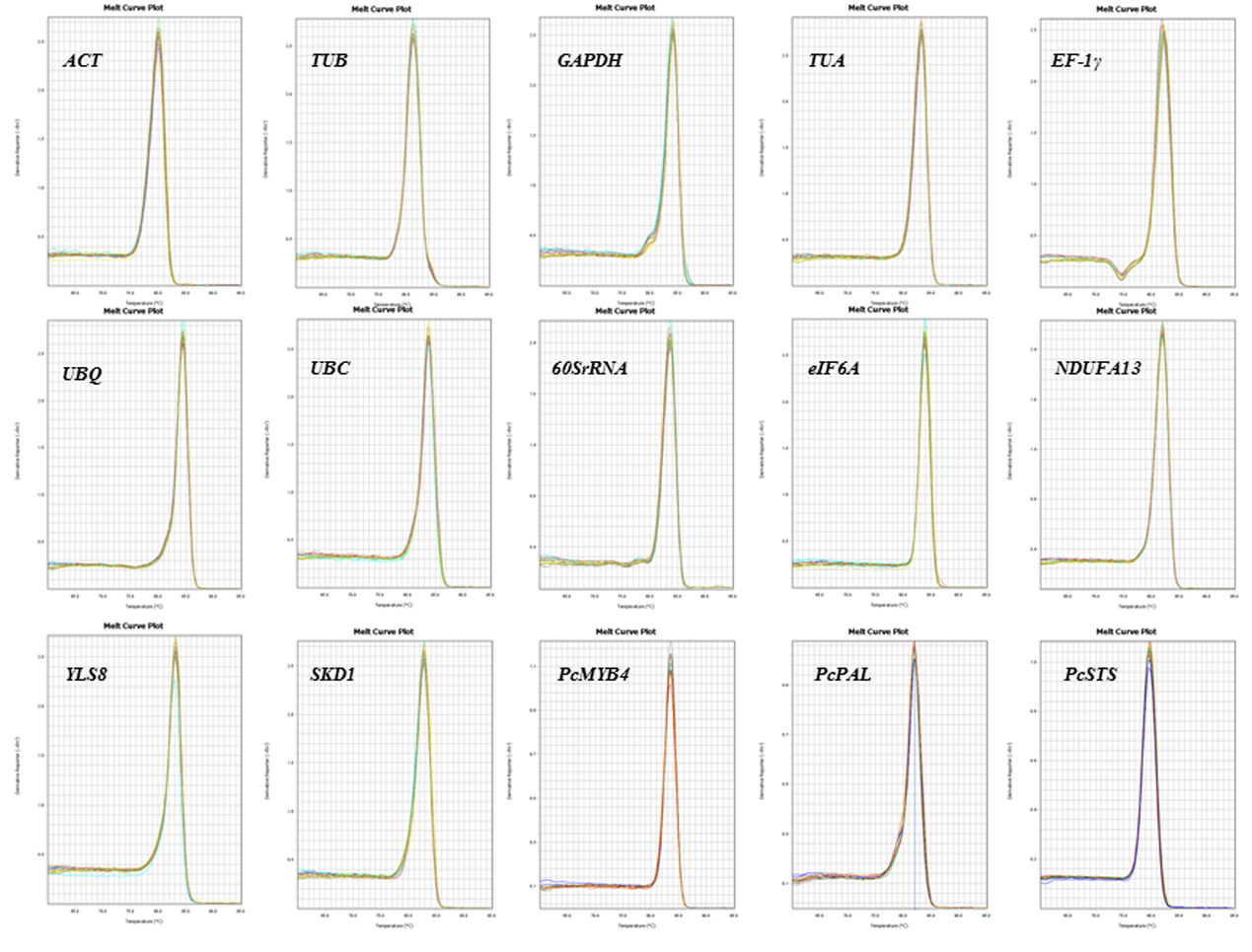


**Figure S2.** Melting curves of 12 candidate reference genes and 3 target genes in *Polygonum cuspidatum.*


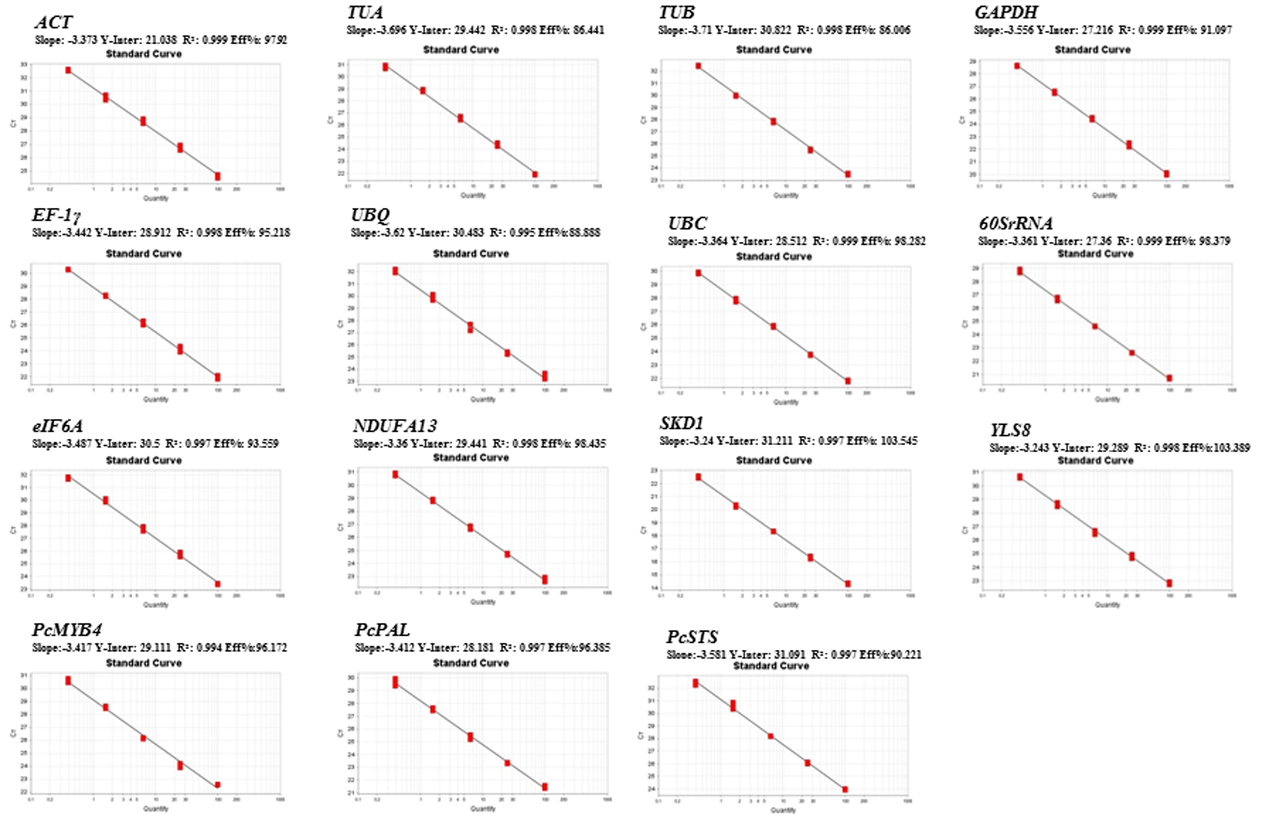


**Figure S3.** Standard curves of 12 candidate reference genes and 3 target genes in *Polygonum cuspidatum.*
