# Supplementary material for: Identification and evaluation of reference genes for quantitative real-time PCR analysis in Polygonum cuspidatum based on transcriptome data
Source: BMC Plant Biol. 2019 Nov 14;19:498. doi: 10.1186/s12870-019-2108-0 (PMC6854638; doi:10.1186/s12870-019-2108-0)
Supplement: Supplementary file 3 — Additional file 3: Table S1. Gene expression stability ranked by △CT, BestKeeper, NormFinder, geNorm and RefFinder under individual condition. Table S2. RPKM values of 3 target genes in Polygonum cuspidatum transcriptome. Table S3. RPKM values of 3 novel genes covering 7 transcriptomes data of Polygonum cuspidatum. [file 12870_2019_2108_MOESM3_ESM.docx]

**Table S1** Gene expression stability ranked by △CT, BestKeeper, NormFinder, geNorm and RefFinder under individual condition.

| Group sets | RefFinder | | △CTnd 7㤀 耀 BestKeeper 7㨀耀geNorm7ăத࿨P耀Genes 7Ā‑耀‑Average of Sta | | BestKeeper | | Normfinder | | geNorm | |
| --- | --- | --- | --- | --- | --- | --- | --- | --- | --- | --- |
|  | Genes | Geomean of Ranking Values | Genes | Average of Standard Deviation | Genes | std dev [+/- CP] | Genes | Stability Value | Genes | Stability Value |
|  | *GAPDH* | 1.63 | *GAPDH* | 0.37 | *YLS8* | 0.17 | *GAPDH* | 0.18 | *TUB* | 0.25 |
|  | *TUB* | 2.21 | *TUB* | 0.38 | *eIF6A* | 0.26 | *TUB* | 0.18 | *GAPDH* | 0.25 |
|  | *YLS8* | 2.28 | *YLS8* | 0.38 | *SKD1* | 0.26 | *YLS8* | 0.19 | *YLS8* | 0.29 |
|  | *SKD1* | 4.56 | *EF-1γ* | 0.38 | *UBC* | 0.27 | *EF-1γ* | 0.21 | *EF-1γ* | 0.30 |
| ABA | *EF-1γ* | 5.03 | *60SrRNA* | 0.39 | *ACT* | 0.28 | *60SrRNA* | 0.23 | *60SrRNA* | 0.31 |
|  | *60SrRNA* | 5.62 | *SKD1* | 0.40 | *TUB* | 0.30 | *SKD1* | 0.26 | *SKD1* | 0.31 |
|  | *eIF6A* | 7.38 | *NDUFA13* | 0.43 | *GAPDH* | 0.32 | *NDUFA13* | 0.28 | *UBQ* | 0.32 |
|  | *UBQ* | 7.97 | *UBQ* | 0.43 | *60SrRNA* | 0.33 | *UBQ* | 0.31 | *NDUFA13* | 0.33 |
|  | *ACT* | 7.98 | *ACT* | 0.46 | *EF-1γ* | 0.35 | *eIF6A* | 0.35 | *ACT* | 0.35 |
|  | *NDUFA13* | 8.10 | *eIF6A* | 0.47 | *UBQ* | 0.35 | *ACT* | 0.36 | *UBC* | 0.37 |
|  | *UBC* | 8.34 | *UBC* | 0.48 | *NDUFA13* | 0.39 | *UBC* | 0.37 | *eIF6A* | 0.38 |
|  | *TUA* | 12.00 | *TUA* | 0.74 | *TUA* | 0.65 | *TUA* | 0.69 | *TUA* | 0.44 |
|  |  |  |  |  |  |  |  |  |  |  |
|  | *GAPDH* | 1.19 | *GAPDH* | 0.29 | *ACT* | 0.23 | *GAPDH* | 0.03 | *NDUFA13* | 0.16 |
|  | *NDUFA13* | 2.06 | *eIF6A* | 0.32 | *NDUFA13* | 0.24 | *NDUFA13* | 0.15 | *GAPDH* | 0.16 |
|  | *SKD1* | 3.72 | *NDUFA13* | 0.33 | *GAPDH* | 0.24 | *eIF6A* | 0.16 | *SKD1* | 0.19 |
|  | *eIF6A* | 3.81 | *SKD1* | 0.33 | *SKD1* | 0.29 | *SKD1* | 0.18 | *YLS8* | 0.22 |
| ETH | *YLS8* | 5.32 | *YLS8* | 0.35 | *UBC* | 0.29 | *YLS8* | 0.22 | *eIF6A* | 0.23 |
|  | *ACT* | 6.45 | *60SrRNA* | 0.38 | *UBQ* | 0.30 | *60SrRNA* | 0.28 | *60SrRNA* | 0.25 |
|  | *60SrRNA* | 6.82 | *TUA* | 0.40 | *eIF6A* | 0.31 | *TUB* | 0.31 | *EF-1γ* | 0.27 |
|  | *UBC* | 7.98 | *TUB* | 0.41 | *YLS8* | 0.34 | *TUA* | 0.31 | *TUA* | 0.29 |
|  | *TUA* | 8.38 | *EF-1γ* | 0.41 | *TUB* | 0.35 | *UBC* | 0.31 | *UBC* | 0.31 |
|  | *TUB* | 8.43 | *UBC* | 0.42 | *TUA* | 0.42 | *EF-1γ* | 0.32 | *TUB* | 0.33 |
|  | *EF-1γ* | 9.32 | *UBQ* | 0.47 | *60SrRNA* | 0.42 | *UBQ* | 0.39 | *UBQ* | 0.35 |
|  | *UBQ* | 9.45 | *ACT* | 0.56 | *EF-1γ* | 0.44 | *ACT* | 0.51 | *ACT* | 0.39 |
|  |  |  |  |  |  |  |  |  |  |  |
|  | *SKD1* | 1.86 | *SKD1* | 0.40 | *ACT* | 0.46 | *SKD1* | 0.07 | *GAPDH* | 0.17 |
|  | *GAPDH* | 2.06 | *GAPDH* | 0.41 | *UBC* | 0.59 | *GAPDH* | 0.07 | *SKD1* | 0.17 |
|  | *NDUFA13* | 3.22 | *NDUFA13* | 0.44 | *eIF6A* | 0.61 | *NDUFA13* | 0.19 | *NDUFA13* | 0.22 |
|  | *eIF6A* | 3.72 | *eIF6A* | 0.44 | *NDUFA13* | 0.62 | *eIF6A* | 0.24 | *eIF6A* | 0.27 |
|  | *UBQ* | 5.44 | *UBQ* | 0.47 | *TUB* | 0.62 | *UBQ* | 0.28 | *UBQ* | 0.28 |
|  | *UBC* | 5.98 | *60SrRNA* | 0.50 | *SKD1* | 0.63 | *60SrRNA* | 0.34 | *60SrRNA* | 0.30 |
| GA | *ACT* | 6.04 | *YLS8* | 0.53 | *UBQ* | 0.64 | *TUB* | 0.39 | *YLS8* | 0.33 |
|  | *60SrRNA* | 6.82 | *UBC* | 0.56 | *YLS8* | 0.67 | *YLS8* | 0.40 | *UBC* | 0.35 |
|  | *YLS8* | 7.48 | *TUB* | 0.57 | *60SrRNA* | 0.69 | *EF-1γ* | 0.41 | *EF-1γ* | 0.38 |
|  | *TUB* | 7.49 | *EF-1γ* | 0.57 | *GAPDH* | 0.69 | *UBC* | 0.44 | *TUB* | 0.42 |
|  | *EF-1γ* | 9.72 | *ACT* | 0.66 | *EF-1γ* | 0.70 | *ACT* | 0.55 | *ACT* | 0.46 |
|  | *TUA* | 12.00 | *TUA* | 1.01 | *TUA* | 0.91 | *TUA* | 0.97 | *TUA* | 0.55 |
|  |  |  |  |  |  |  |  |  |  |  |
|  | *60SrRNA* | 1.57 | *60SrRNA* | 0.27 | *SKD1* | 0.29 | *60SrRNA* | 0.10 | *60SrRNA* | 0.16 |
|  | *YLS8* | 1.86 | *NDUFA13* | 0.27 | *YLS8* | 0.29 | *NDUFA13* | 0.12 | *YLS8* | 0.16 |
|  | *NDUFA13* | 2.63 | *eIF6A* | 0.27 | *UBC* | 0.30 | *eIF6A* | 0.12 | *NDUFA13* | 0.18 |
| MeJA | *eIF6A* | 4.43 | *YLS8* | 0.28 | *NDUFA13* | 0.32 | *YLS8* | 0.12 | *eIF6A* | 0.19 |
|  | *UBC* | 4.82 | *EF-1γ* | 0.31 | *60SrRNA* | 0.32 | *EF-1γ* | 0.20 | *UBC* | 0.20 |
|  | *SKD1* | 5.47 | *UBC* | 0.32 | *TUB* | 0.32 | *UBC* | 0.21 | *EF-1γ* | 0.22 |
|  | *EF-1γ* | 6.06 | *GAPDH* | 0.33 | *GAPDH* | 0.34 | *GAPDH* | 0.23 | *SKD1* | 0.24 |
|  | *GAPDH* | 7.24 | *SKD1* | 0.33 | *eIF6A* | 0.34 | *SKD1* | 0.24 | *GAPDH* | 0.25 |
|  | *TUB* | 8.41 | *UBQ* | 0.36 | *EF-1γ* | 0.35 | *UBQ* | 0.28 | *UBQ* | 0.26 |
|  | *UBQ* | 9.46 | *TUB* | 0.43 | *ACT* | 0.38 | *TUB* | 0.37 | *TUB* | 0.29 |
|  | *ACT* | 10.74 | *ACT* | 0.43 | *UBQ* | 0.40 | *ACT* | 0.38 | *ACT* | 0.32 |
|  | *TUA* | 12.00 | *TUA* | 0.45 | *TUA* | 0.49 | *TUA* | 0.40 | *TUA* | 0.34 |
|  |  |  |  |  |  |  |  |  |  |  |
|  | *NDUFA13* | 1.57 | *NDUFA13* | 0.45 | *ACT* | 0.19 | *UBQ* | 0.14 | *NDUFA13* | 0.24 |
|  | *UBQ* | 2.51 | *UBQ* | 0.46 | *SKD1* | 0.31 | *NDUFA13* | 0.15 | *SKD1* | 0.24 |
|  | *SKD1* | 2.78 | *GAPDH* | 0.47 | *NDUFA13* | 0.33 | *GAPDH* | 0.23 | *GAPDH* | 0.28 |
|  | *GAPDH* | 3.22 | *eIF6A* | 0.47 | *GAPDH* | 0.41 | *eIF6A* | 0.24 | *UBQ* | 0.30 |
|  | *eIF6A* | 5.26 | *YLS8* | 0.49 | *UBQ* | 0.41 | *SKD1* | 0.28 | *YLS8* | 0.32 |
| SA | *YLS8* | 5.48 | *SKD1* | 0.50 | *YLS8* | 0.44 | *YLS8* | 0.29 | *eIF6A* | 0.33 |
|  | *ACT* | 5.90 | *UBC* | 0.57 | *UBC* | 0.53 | *UBC* | 0.43 | *UBC* | 0.36 |
|  | *UBC* | 7.00 | *EF-1γ* | 0.59 | *eIF6A* | 0.60 | *EF-1γ* | 0.45 | *EF-1γ* | 0.39 |
|  | *EF-1γ* | 8.46 | *60SrRNA* | 0.60 | *TUB* | 0.62 | *60SrRNA* | 0.47 | *60SrRNA* | 0.40 |
|  | *60SrRNA* | 9.46 | *TUB* | 0.67 | *EF-1γ* | 0.71 | *TUB* | 0.50 | *ACT* | 0.45 |
|  | *TUB* | 9.97 | *ACT* | 0.67 | *60SrRNA* | 0.79 | *ACT* | 0.53 | *TUB* | 0.49 |
|  | *TUA* | 12.00 | *TUA* | 1.08 | *TUA* | 0.87 | *TUA* | 1.02 | *TUA* | 0.58 |
|  |  |  |  |  |  |  |  |  |  |  |
|  | *YLS8* | 1.32 | *YLS8* | 0.28 | *TUB* | 0.25 | *YLS8* | 0.03 | *NDUFA13* | 0.11 |
|  | *NDUFA13* | 2.21 | *NDUFA13* | 0.30 | *GAPDH* | 0.29 | *EF-1γ* | 0.11 | *YLS8* | 0.11 |
|  | *TUB* | 3.50 | *EF-1γ* | 0.30 | *YLS8* | 0.32 | *NDUFA13* | 0.12 | *60SrRNA* | 0.12 |
|  | *EF-1γ* | 3.83 | *60SrRNA* | 0.31 | *NDUFA13* | 0.34 | *60SrRNA* | 0.15 | *EF-1γ* | 0.13 |
|  | *60SrRNA* | 4.28 | *TUB* | 0.33 | *eIF6A* | 0.36 | *GAPDH* | 0.15 | *TUB* | 0.17 |
| Salt | *GAPDH* | 4.36 | *GAPDH* | 0.33 | *ACT* | 0.36 | *TUB* | 0.19 | *GAPDH* | 0.19 |
|  | *UBQ* | 7.65 | *UBQ* | 0.39 | *60SrRNA* | 0.37 | *UBQ* | 0.28 | *UBQ* | 0.22 |
|  | *eIF6A* | 8.61 | *SKD1* | 0.43 | *UBC* | 0.38 | *SKD1* | 0.33 | *SKD1* | 0.26 |
|  | *SKD1* | 8.66 | *UBC* | 0.43 | *EF-1γ* | 0.38 | *UBC* | 0.35 | *UBC* | 0.29 |
|  | *UBC* | 8.74 | *eIF6A* | 0.47 | *UBQ* | 0.39 | *ACT* | 0.40 | *eIF6A* | 0.31 |
|  | *ACT* | 9.23 | *ACT* | 0.49 | *SKD1* | 0.44 | *eIF6A* | 0.40 | *ACT* | 0.34 |
|  | *TUA* | 12.00 | *TUA* | 0.65 | *TUA* | 0.56 | *TUA* | 0.60 | *TUA* | 0.39 |
|  |  |  |  |  |  |  |  |  |  |  |
|  | *UBC* | 1.68 | *UBC* | 0.43 | *60SrRNA* | 0.56 | *EF-1γ* | 0.05 | *TUB* | 0.14 |
|  | *EF-1γ* | 2.34 | *EF-1γ* | 0.43 | *YLS8* | 0.65 | *UBC* | 0.12 | *UBC* | 0.14 |
|  | *TUB* | 3.08 | *TUB* | 0.46 | *NDUFA13* | 0.68 | *TUB* | 0.22 | *EF-1γ* | 0.21 |
|  | *60SrRNA* | 3.81 | *NDUFA13* | 0.49 | *UBC* | 0.74 | *NDUFA13* | 0.24 | *eIF6A* | 0.25 |
|  | *NDUFA13* | 4.28 | *YLS8* | 0.49 | *EF-1γ* | 0.74 | *60SrRNA* | 0.27 | *YLS8* | 0.27 |
| UV | *YLS8* | 4.33 | *eIF6A* | 0.50 | *GAPDH* | 0.78 | *eIF6A* | 0.29 | *60SrRNA* | 0.31 |
|  | *eIF6A* | 6.31 | *60SrRNA* | 0.51 | *UBQ* | 0.79 | *YLS8* | 0.30 | *NDUFA13* | 0.34 |
|  | *GAPDH* | 7.44 | *GAPDH* | 0.56 | *SKD1* | 0.81 | *GAPDH* | 0.39 | *GAPDH* | 0.38 |
|  | *ACT* | 9.00 | *ACT* | 0.58 | *ACT* | 0.82 | *ACT* | 0.41 | *ACT* | 0.40 |
|  | *SKD1* | 9.46 | *SKD1* | 0.62 | *TUB* | 0.83 | *SKD1* | 0.52 | *SKD1* | 0.43 |
|  | *UBQ* | 9.82 | *UBQ* | 0.76 | *eIF6A* | 0.86 | *UBQ* | 0.71 | *UBQ* | 0.48 |
|  | *TUA* | 12.00 | *TUA* | 1.05 | *TUA* | 1.12 | *TUA* | 1.02 | *TUA* | 0.57 |
|  |  |  |  |  |  |  |  |  |  |  |
|  | *60SrRNA* | 1.57 | *60SrRNA* | 0.25 | *NDUFA13* | 0.27 | *60SrRNA* | 0.10 | *60SrRNA* | 0.11 |
|  | *SKD1* | 2.11 | *SKD1* | 0.26 | *GAPDH* | 0.28 | *SKD1* | 0.12 | *SKD1* | 0.11 |
|  | *GAPDH* | 3.31 | *GAPDH* | 0.28 | *ACT* | 0.31 | *ACT* | 0.17 | *GAPDH* | 0.14 |
|  | *ACT* | 3.46 | *ACT* | 0.28 | *UBQ* | 0.31 | *GAPDH* | 0.18 | *ACT* | 0.16 |
|  | *NDUFA13* | 4.23 | *eIF6A* | 0.29 | *SKD1* | 0.34 | *eIF6A* | 0.18 | *NDUFA13* | 0.17 |
| Cold | *eIF6A* | 5.79 | *TUA* | 0.29 | *60SrRNA* | 0.36 | *UBC* | 0.19 | *UBC* | 0.19 |
|  | *UBC* | 6.48 | *UBC* | 0.30 | *UBC* | 0.38 | *TUA* | 0.19 | *eIF6A* | 0.22 |
|  | *UBQ* | 6.84 | *NDUFA13* | 0.31 | *eIF6A* | 0.39 | *NDUFA13* | 0.22 | *TUA* | 0.24 |
|  | *TUA* | 7.42 | *UBQ* | 0.33 | *TUA* | 0.42 | *UBQ* | 0.24 | *UBQ* | 0.25 |
|  | *EF-1γ* | 10.24 | *EF-1γ* | 0.34 | *YLS8* | 0.42 | *EF-1γ* | 0.26 | *EF-1γ* | 0.26 |
|  | *TUB* | 11.24 | *TUB* | 0.40 | *EF-1γ* | 0.47 | *TUB* | 0.35 | *TUB* | 0.28 |
|  | *YLS8* | 11.47 | *YLS8* | 0.49 | *TUB* | 0.54 | *YLS8* | 0.45 | *YLS8* | 0.32 |
|  |  |  |  |  |  |  |  |  |  |  |
|  | *EF-1γ* | 2.00 | *EF-1γ* | 0.62 | *TUB* | 0.61 | *EF-1γ* | 0.20 | *ACT* | 0.26 |
|  | *NDUFA13* | 3.60 | *NDUFA13* | 0.65 | *eIF6A* | 0.70 | *60SrRNA* | 0.34 | *GAPDH* | 0.26 |
|  | *60SrRNA* | 3.66 | *60SrRNA* | 0.67 | *EF-1γ* | 0.78 | *UBC* | 0.37 | *NDUFA13* | 0.31 |
|  | *ACT* | 3.94 | *UBC* | 0.67 | *YLS8* | 0.78 | *NDUFA13* | 0.38 | *EF-1γ* | 0.36 |
| Heat | *GAPDH* | 4.64 | *ACT* | 0.75 | *60SrRNA* | 0.84 | *UBQ* | 0.49 | *UBC* | 0.41 |
|  | *UBC* | 4.82 | *GAPDH* | 0.77 | *UBQ* | 0.93 | *ACT* | 0.55 | *60SrRNA* | 0.46 |
|  | *TUB* | 5.03 | *UBQ* | 0.77 | *NDUFA13* | 0.99 | *GAPDH* | 0.59 | *SKD1* | 0.51 |
|  | *UBQ* | 6.40 | *TUB* | 0.83 | *ACT* | 1.00 | *TUB* | 0.60 | *UBQ* | 0.55 |
|  | *eIF6A* | 7.67 | *TUA* | 0.84 | *TUA* | 1.07 | *TUA* | 0.63 | *TUA* | 0.59 |
|  | *YLS8* | 7.95 | *SKD1* | 0.86 | *UBC* | 1.07 | *SKD1* | 0.70 | *TUB* | 0.65 |
|  | *TUA* | 9.24 | *YLS8* | 1.05 | *GAPDH* | 1.08 | *YLS8* | 0.94 | *YLS8* | 0.74 |
|  | *SKD1* | 9.57 | *eIF6A* | 1.12 | *SKD1* | 1.33 | *eIF6A* | 1.02 | *eIF6A* | 0.80 |
|  |  |  |  |  |  |  |  |  |  |  |
|  | *NDUFA13* | 1.32 | *NDUFA13* | 0.20 | *ACT* | 0.29 | *NDUFA13* | 0.08 | *ACT* | 0.13 |
|  | *ACT* | 1.73 | *TUB* | 0.21 | *UBC* | 0.33 | *TUB* | 0.08 | *NDUFA13* | 0.13 |
|  | *TUB* | 3.13 | *ACT* | 0.23 | *NDUFA13* | 0.38 | *ACT* | 0.14 | *UBQ* | 0.14 |
|  | *UBQ* | 4.16 | *UBQ* | 0.24 | *UBQ* | 0.39 | *UBQ* | 0.15 | *TUB* | 0.16 |
| Drought | *YLS8* | 4.86 | *YLS8* | 0.24 | *TUB* | 0.42 | *YLS8* | 0.15 | *YLS8* | 0.17 |
|  | *EF-1γ* | 6.44 | *eIF6A* | 0.24 | *EF-1γ* | 0.42 | *eIF6A* | 0.15 | *eIF6A* | 0.19 |
|  | *eIF6A* | 6.64 | *EF-1γ* | 0.25 | *YLS8* | 0.42 | *EF-1γ* | 0.17 | *EF-1γ* | 0.20 |
|  | *UBC* | 7.18 | *60SrRNA* | 0.26 | *GAPDH* | 0.46 | *60SrRNA* | 0.19 | *60SrRNA* | 0.21 |
|  | *GAPDH* | 8.74 | *GAPDH* | 0.27 | *eIF6A* | 0.46 | *GAPDH* | 0.20 | *GAPDH* | 0.22 |
|  | *60SrRNA* | 8.85 | *TUA* | 0.30 | *SKD1* | 0.47 | *TUA* | 0.25 | *TUA* | 0.23 |
|  | *TUA* | 10.24 | *UBC* | 0.31 | *TUA* | 0.48 | *UBC* | 0.27 | *UBC* | 0.24 |
|  | *SKD1* | 11.47 | *SKD1* | 0.35 | *60SrRNA* | 0.50 | *SKD1* | 0.30 | *SKD1* | 0.26 |
|  |  |  |  |  |  |  |  |  |  |  |
|  | *NDUFA13* | 1.57 | *NDUFA13* | 0.31 | *GAPDH* | 0.92 | *ACT* | 0.06 | *NDUFA13* | 0.10 |
|  | *EF-1γ* | 2.45 | *EF-1γ* | 0.32 | *NDUFA13* | 1.03 | *NDUFA13* | 0.10 | *EF-1γ* | 0.10 |
|  | *ACT* | 2.63 | *ACT* | 0.32 | *EF-1γ* | 1.07 | *60SrRNA* | 0.10 | *60SrRNA* | 0.13 |
|  | *60SrRNA* | 3.13 | *60SrRNA* | 0.32 | *60SrRNA* | 1.08 | *EF-1γ* | 0.13 | *ACT* | 0.16 |
| Different tissues | *GAPDH* | 4.43 | *eIF6A* | 0.37 | *TUB* | 1.11 | *eIF6A* | 0.22 | *TUB* | 0.19 |
|  | *TUB* | 5.69 | *TUB* | 0.38 | *ACT* | 1.19 | *UBQ* | 0.26 | *GAPDH* | 0.21 |
|  | *eIF6A* | 6.32 | *UBQ* | 0.39 | *TUA* | 1.23 | *TUB* | 0.27 | *UBQ* | 0.24 |
|  | *UBQ* | 7.17 | *GAPDH* | 0.42 | *eIF6* | 1.24 | *GAPDH* | 0.32 | *eIF6A* | 0.27 |
|  | *YLS8* | 9.24 | *YLS8* | 0.46 | *UBQ* | 1.30 | *YLS8* | 0.38 | *YLS8* | 0.31 |
|  | *TUA* | 10.49 | *UBC* | 0.54 | *YLS8* | 1.34 | *SKD1* | 0.47 | *UBC* | 0.35 |
|  | *UBC* | 10.49 | *SKD1* | 0.54 | *UBC* | 1.40 | *UBC* | 0.50 | *SKD1* | 0.38 |
|  | *SKD1* | 10.98 | *TUA* | 0.57 | *SKD1* | 1.46 | *TUA* | 0.54 | *TUA* | 0.41 |
|  |  |  |  |  |  |  |  |  |  |  |

**Table S2** RPKM values of 3 target genes in *Polygonum cuspidatum* transcriptome.

| Gene name | Leaf-Mean | Stem-Mean | Root-Mean | UV-2h-leaf-Mean | UV-8H-leaf-Mean |
| --- | --- | --- | --- | --- | --- |
| *PcMYB4* | 12.0260 | 4.1014 | 8.9417 | 2.9294 | 3.2112 |
| *PcPAL* | 55.9571 | 309.2043 | 240.6385 | 386.9797 | 284.5269 |
| *PcSTS* | 15.3464 | 14.0594 | 8.7344 | 20.0061 | 3.6192 |

**Table S3** RPKM values of 3 novel genes covering 7 transcriptomes data of *Polygonum cuspidatum*.

| Gene name | Leaf-Mean | Stem-Mean | Root-Mean | MeJA-8h-leaf-Mean | MeJA-16h-leaf-Mean | UV-2h-leaf-Mean | UV-8h-leaf-Mean | Average value | *CV* value |
| --- | --- | --- | --- | --- | --- | --- | --- | --- | --- |
| *SKD1* | 127.7549 | 126.1010 | 152.0393 | 142.0997 | 155.0213 | 123.1688 | 146.5443 | 138.9613 | 0.0946 |
| *YLS8* | 115.2649 | 109.1016 | 126.9067 | 104.2979 | 123.4727 | 132.2242 | 134.8068 | 120.8678 | 0.0962 |
| *NDUFA13* | 107.2619 | 126.2193 | 118.8914 | 116.5564 | 127.6877 | 110.7795 | 85.7368 | 113.3047 | 0.1258 |

Note：Coefficient of variation (*CV*) was calculated for every gene and listed last. Data are unpublished.
