# Supplementary material for: Identification and evaluation of reference genes for quantitative real-time PCR analysis in Polygonum cuspidatum based on transcriptome data
Source: BMC Plant Biol. 2019 Nov 14;19:498. doi: 10.1186/s12870-019-2108-0 (PMC6854638; doi:10.1186/s12870-019-2108-0)
Supplement: Supplementary file 4 — Additional file 4. cDNA sequences of 12 candidate reference genes and 3 target genes. Coding sequence (CDS) were marked green. [file 12870_2019_2108_MOESM4_ESM.docx]

>*ACT* GGTTGGCGTGCGAACTTGCGAATTGCTTTGTCGGCGAGTGTGCGGGCTCAAGTAATTAAATTAGAAATTAATTAGAAAAAGTAGAGGGTGGGGCCTGAGGGTCACTGACCCTCCGGGTCACATAATAAAATTCCCTTCGTAGGGTGAGCAGACAACTGAAACCCATACAACACAGAGAAGAACAAAGAGAAGTGCGTGCCTCTGCCGCCTTCTCCTTTGCAAGTGAAGGACTGCTCCGCGCGCGTTCTCTTACTCTTACCAAAGTTCTTTGTCTCCCCGATCTATAGAAGGTAACTAGTAATGGCTGACGAGGAAATCCAACCCCTTGTTTGTGACAATGGTACTGGTATGGTGAAGGCTGGGTTTGCAGGAGATGATGCTCCTAGGGCTGTGTTCCCCAGTATTGTGGGTAGGCCAAGACACACTGGTGTTATGGTTGGCATGGGGCAGAAGGATGCTTACGTTGGTGACGAGGCTCAATCCAAAAGAGGTATTCTGACCTTGAAATACCCCATTGAGCACGGTATTGTCAGCAACTGGGATGACATGGAAAAGATCTGGCATCACACCTTCTACAACGAGCTTCGTGTTGCTCCTGAGGAACATCCAGTGCTTCTCACTGAGGCTCCCCTCAACCCTAAGGCCAACAGGGAGAAGATGACTCAAATCATGTTTGAGACATTCAATGTCCCTGCCATGTATGTTGCTATCCAGGCTGTTCTATCTCTCTATGCTAGTGGTCGTACAACTGGTATTGTGCTGGATTCTGGTGATGGTGTGAGTCACACTGTCCCCATTTATGAAGGTTATGCTCTCCCCCACGCTATCCTTCGTCTGGACCTTGCTGGACGTGACCTTACTGATTCCCTCATGAAGATCCTTACTGAAAGAGGGTACATGTTCACCACCACTGCTGAACGGGAAATTGTCCGTGACATTAAGGAAAAGCTTGCGTATGTTGCTCTTGACTATGAGCAAGAGCTTGAAACCGCCAAGAGCAGCTCAGCCATTGAGAAGAACTATGAGCTTCCTGATGGACAAGTCATTACAATTGGTGCTGAGAGGTTCAGATGCCCTGAGGTTCTCTTCCAGCCGTCTATGATTGGAATGGAAGCTGCAGGAATTCACGAGACTACCTACAACTCCATCATGAAGTGTGATGTCGATATTAGGAAGGACTTGTACGGTAACATTGTGCTCAGTGGAGGTTCAACTATGTTCCCTGGTATAGCTGACAGGATGAGCAAGGAGATCACTGCCCTTGCTCCAAGCAGCATGAAGATCAAGGTTGTGGCTCCTCCTGAGAGGAAATACAGTGTCTGGATTGGAGGATCCATCCTTGCATCTCTCAGCACCTTCCAGCAGATGTGGATATCCAAGGGAGAATATGACGAATCCGGTCCAGCCATTGTCCACAGGAAGTGCTTCTAAGTACCAGTTCCTCCTCCACATGTTTTGTCATGCATCATGCTGATGACTCCATTGGGTGGAGAAAAAAGGGTCTTTGAAGAAATATATCTTCTGGTATTTTAAGTGTCATGTTCGTTTACATTTAGTGGTTTATATTATAAACATGTGTTGCGTCTTATTTTATGGGTAAGCCAGTAATGAGCCCTATTGCGTCTTATTTTCCGGGTTCAGCAAGTCTTGTAGGACGAGTCAAAGAGTGATTTGTGATGCTTTTTCTCTTATTTCACTGCTCAGATTTTGAAGGTTCTCTTTTCTGGCTGT

>*TUA*

CTCATTTCTCATTTCTCAGTGCAGAGCGTCTTGATAAACGCTCTTCTTTTTCATTTCGCCTTCAGTTGTTCTCTTCCTTCGCCAAATTCTCGCTTTTTCTCGCTTTCGAAAATGAGAGAGTGCATCTCGATCCACATTGGTCAGGCCGGTATCCAGGTCGGCAATGCCTGCTGGGAGCTTTACTGTCTCGAGCACGGTATCCAGCCTGATGGCCAGATGCCAAGTGACAAAACTGTTGGTGGAGGTGATGATGCCTTCAACACCTTCTTCAGTGAGACTGGTGCTGGGAAGCACGTTCCTCGTGCTGTCTTTGTAGATCTTGAGCCTACTGTCATTGATGAAGTAAGGACTGGAACATACAGACAACTCTTCCACCCTGAGCAACTCATCAGTGGGAAAGAAGATGCTGCTAACAACTTTGCTCGTGGACACTACACAATTGGAAAGGAGATTGTGGATCTCTGCTTGGACAGGATCAGGAAGCTGGCTGACAACTGCACCGGTCTTCAAGGTTTCCTTGTATTCAATGCTGTTGGTGGTGGCACTGGATCTGGTCTGGGGTCACTTCTCCTAGAGAGGCTCTCAGTTGACTATGGAAAGAAGTCAAAGTTGGGTTTTACTGTCTACCCATCTCCACAAGTGTCAACCTCTGTTGTTGAGCCCTACAACAGTGTTCTATCCACCCACTCCCTCCTTGAGCACACTGATGTCTCTGTTCTCCTTGACAATGAAGCCATCTATGACATCTGCAGGCGCTCCCTGGACATTGAGCGCCCCACATACACCAACCTCAACCGTCTTGTCTCTCAGGTCATTTCTTCCCTGACAGCATCTCTGAGATTTGATGGAGCCCTGAATGTGGATGTGAATGAGTTCCAGACCAACCTTGTTCCCTACCCAAGGATCCACTTCATGCTTTCTTCCTATGCTCCAGTCATCTCTGCTGAGAAGGCCTACCATGAGCAGCTCTCAGTGGCTGAGATCACCAACAGTGCCTTTGAACCCTCATCCATGATGGCCAAGTGTGACCCACGTCACGGGAAGTACATGGCTTGCTGTCTGATGTACCGTGGTGATGTTGTGCCCAAGGATGTCAATGCTGCTGTTGCTACTATCAGGACTAAGCGCACCATCCAGTTTGTCGACTGGTGCCCTACTGGCTTCAAGTGTGGTATCAACTACCAGCCACCAACTGTGGTTCCTGGTGGTGACCTTGCCAAGGTTCAGAGGGCCGTTTGCATGATTTCCAACTCCACCAGTGTTGCTGAGGTGTTCTCTCGCATTGACCACAAGTTTGATCTGATGTATGCTAAGCGTGCCTTTGTTCACTGGTATGTGGGTGAGGGTATGGAGGAAGGAGAGTTCTCTGAAGCTCGTGAGGACCTTGCTGCTCTTGAGAAGGACTATGAAGAGGTTGGTGCTGAAGGTGTTGAGGGTGAGGATGATGATGGTGAAGAGTACTGATGAATTTCCTAGTATTGTGTCTTTTAAAATCTGAGATGTGTTTTTTTTCCCTAAATATGGAGCTGTATGATCTCCATGCG

>*TUB*

TAAACTCTTCTCTTCTCTCCCCAACCAGTACTTTCTCTCTCCTCCATAGCCTCCATCGAAGATGCGTGAGATCCTTCACATCCAGGGTGGTCAGTGCGGGAACCAGATTGGGGCCAAGTTCTGGGAGGTGGTGTGCGCGGAGCACGGCATCGACTCCACTGGTCGCTACAATGGCGACTCAGAGCTTCAGCTTGAGAGGGTGAATGTGTACTACAACGAGGCCAGCTGTGGCCGCTTCGTCCCCAGGGCGGTGCTCATGGACTTGGAGCCTGGAACCATGGACAGTTTAAGATCTGGTCCCTACGGTCAGATCTTCAGGCCTGATAACTTCGTCTTTGGTCAGTCCGGTGCCGGCAATAACTGGGCTAAGGGGCACTATACCGAGGGTGCTGAGCTCATCGATTCTGTGCTTGATGTTGTGAGGAAGGAGGCCGAGAACTGTGATTGCTTGCAAGGATTCCAAGTTTGCCATTCACTTGGTGGAGGTACAGGTTCTGGTATGGGAACCCTTCTTATTTCGAAAATTCGGGAGGAGTATCCTGACCGGATGATGTTGACATTCTCTGTCTTCCCTTCACCGAAAGTATCCGACACTGTTGTTGAGCCTTACAATGCCACTTTATCAGTTCATCAGCTTGTTGAAAATGCTGATGAGTGTATGGTTTTGGACAATGAGGCTCTCTATGACATTTGCTTCCGGACTCTGAAGCTTTCAACCCCATCCTTTGGGGATCTCAACCACCTCATCTCCGTCACCATGAGTGGTGTAACATGCTGTTTACGTTTCCCTGGCCAACTCAACTCCGACCTCCGTAAATTAGCAGTAAATCTCATCCCCTTCCCACGTCTCCACTTCTTCATGGTTGGCTTTGCCCCCTTAACCTCAAGAGGCTCCCAACAATACAGAGCTCTAACCGTCCCTGAACTCACCCAACAAATGTGGGACGCGAAAAACATGATGTGTGCAGCCGACCCACGCCATGGCAGGTACCTAACTGCTTCAGCTGTGTTCAGAGGCAAAATGAGCACAAAGGAAGTTGATGAGCAAATGATCAACATCCAGAACAAGAACTCGTCTTACTTTGTTGAGTGGATCCCGAATAATGTAAAGTCGAGCGTTTGTGATATTCCTCCAAGGGGATTGCAAATGTCTTCTACTTTCATTGGGAATTCGACGTCCATTCAAGAGATGTTTAGGAGAGTGAGCGAGCAGTTTACTGCCATGTTCAGGAGGAAGGCTTTCCTGCACTGGTACACGGGTGAAGGAATGGATGAGATGGAGTTTACTGAGGCGGAGAGCAATATGAATGATCTGGTGTCTGAGTATCAGCAGTACCAGGATGCAACTGCTGATGAGGAGGGTGAGTACGAGGATGAGGAGGAGGACGACGAGGAACAGTTTGCATGAAAGACTTCCTAGAATCTCATCTGGCTGTGTGCAAGTGATATATTGAGTTGTCTTTGTATGTTGAGAAGGTGTTTTGGTATGGTGAATCTTGTGTTTGAAGTTTCTGTATTGTGGTTTCTGCAGATTCGTCTCCCAAGTGCTTGTAATCATATTTTCCATCATACCCTTCTATATTCCGTCATTTACGTTGCTTTGTTCTATTAATTTTGGCGCCCTGGTATCTTGAAGCAGTGTGTTGTGATAAATCCCCGAAGAATATTGCTGCAGCTAGACAGAACAAAAACGGGTTTGGGTGGGATG

>*GAPDH*

TGGGTTCCTGCAACCGAAAACAGTCACTGGTGACTGACCAAAGAGAAGAGTCTTCTCATCCTGGACCTTGACGTCGTGGTGCTTCCAGTGACCGTGGACAGTGTCATACTTGAACATGTATGTCATGTATTCGGTGGTGATGAAGGGGTCGTTGACGGCAACGAGCTCCACATCCTCCCTTGAAAGAATCACCCTAGCGACCAAACGGCCGATTCTTCCGAAACCGTTGATTCCGATCTTGATCTTTGCCATDHTCATCTTCTCAAATTTCATCTCTGCTCTCTCCTCTCACTCTCGTCAATGGCAAAGATCAAGATCGGAATCAACGGTTTCGGAAGAATCGGCCGTTTGGTCGCTAGGGTGATTCTTTCAAGGGAGGATGTGGAGCTCGTTGCCGTCAACGACCCCTTCATCACCACCGAATACATGACATACATGTTCAAGTATGACACTGTCCACGGTCACTGGAAGCACCACGACGTCAAGGTCCAGGATGAGAAGACTCTTCTCTTTGGTCAGTCACCAGTGACTGTTTTCGGTTGCAGGAACCCAGAGGAGATCCCATGGGCTCAGACTGGTGCTGACTTCGTTGTTGAGTCCACTGGAGTCTTCACCGACAAGGACAAGGCTGCTGCTCACTTGAAGGGTGGTGCTAAGAAGGTCGTCATCTCTGCACCTAGCAAGGATGCACCTATGTTTGTTGTTGGTGTTAATGAGCATGAGTACAAGCCTGAGCTTGACATTGTCTCCAATGCCAGTTGCACAACCAACTGCCTTGCTCCCTTGGCTAAGGTTATCAATGACAACTTTGGCATTGTTGAGGGCCTAATGACCACAGTTCACGCAATGACCGCCACACAGAAGACTGTTGATGGTCCATCAATGAAGGACTGGAGAGGTGGAAGGGCTGCTTCATTCAACATCATTCCCAGCAGCACTGGAGCTGCTAAGGCTGTTGGCAAGGTGTTGCCTGCTTTGAATGGAAAGTTGACCGGAATGGCATTCCGTGTCCCAACTGTTGATGTGTCTGTGGTTGACCTCACTGTCAGACTTGAGAAGGCTGCCAGCTACGAGGACATTAAGCGTGCCATCAAGGCGGCATCTGAGGGCAAGATGAAGGGAATTATGGGTTACACTGACGAGGATCTTGTTTCTACTGACTTTATCGGTGACAACAGATCAAGCATCTTTGATGCCAAAGCTGGTATTGCTTTGAATGAGAATTTTGTCAAGCTTGTGTCCTGGTATGACAACGAAATGGGTTACAGTACCCGTGTCGTTGACTTGATCTGCCACATGGCCAAGTCCCTGTAAGCTACTCCAACAAGATGGAGTCTCACAGTTGGCTCCATCGGGTCATTGCCTGAGTTTCTTATGTATCTTTGATTTTGAGTAGTGAGGCTCTGTTAAAACTGATGAATAAAGCAGAGATGTTACAGTTGTTTGGTTTGGTTTGTTCCAGTTTCTCAGTTTATTACCAGCACTTGTCTAAGAACTACCACAAAGATCTGGCAAGGGTATTTGTACTTTTGTAGCAAATATTCGGCTAATATGACTCCTTTTGTGTGTTATGCATTATATAGTAGTCTGTTAATTTCTCACTGGCTTTTTTGA

>*EF-1γ*

AAAAACCCTCCTCTCTCTATAGCCGCTGTCCTCTTCTCCGGTGCCCGAGTCTTCTGCGTCCAACTGTCCAAGAGAATCTATCTTCGTCGTAGAGCTTATCCGGCGCAAACATGGCTCTGTTTTTGCACTCCGGAAGCAACAACAAGAATGCCTACAAGGCTCTCATTGCTGCTGAATACACTGGTGTTCGGGTTGAGTTAGCCAAGGATTTTAAAATGGGAGAATCCAACAAAACTCCAGATTTCCTTAAGATGAACCCTATCGGAAAGGTTCCTGTACTGGAAACACCCGAGGGGCCTGTCTTTGAGAGCAATGCTATTGCACGCTATGTGGCTCGCTTGAATGCCGACAGTACACTCTATGGGTCATCCCTGATTGATTACGCTCATGTTGAGCAGTGGATGGATTTTGCATCCATGGAGATTGATGCAAATATTGCCAGGTGGCTATTTCCCCGTCTTGGCTTTATTGCCCACCTTCCCCTGGCTGAGGAATTTGGAATTTCTCAATTGAAGAGGTCTTTGGAAGCATTGAACACACATCTTGCTTCAAACACATTCCTGGTTGGACACGGTGTTACATTGGCTGACATTGTCATGACATGTAACTTGTATCTTGGGTTTAGCCGCATCATGACCAAAACTTTCACCAAGGATTTCCCACATGTTGAGAGATACTTCTGGACCATGGTTAACCAACCGAATTTCAAGAAGGTTTTGGGTGAAGTGAAACAAGCAACATCCGTTCCAGCAATTGAAAAGAAGCAGGCACAACCCGCCAAGCAGAAGCCTAAGGAAGAACCAAAGAAGGCAGCACCAAAACCCAAAGAAGAAGCTCCTGCTGAAGAGGAGGAGGAGGCTCCCAAGCCTAAACCAAAGAATCCTCTAGATCTTCTTCCTCCTAGTAAGATGATTCTAGATGAGTGGAAGAGGCTCTACTCTAACACTAAATCCAACTTCCGTGAGGTTGCTATCAAAGGATTCTGGGATATGTATGATCCTGAAGGATACTCTTTGTGGTTCTGTAACTACAAGTACAATGAGGAGAACACCGTCTCATTTGTTACTCTAAACAAAGTTGGTGGATTCCTGCAAAGAATGGATTTGGCGCGCAAGTATGCCTTTGGAAAGATGTTGGTGATTGGCTCTGAACCTCCATTTAAGGTCAAGGGATTGTGGCTATTCCGTGGACAAGAAATTCCCAAGTTTGTGATGGATGAGTGCTATGACATGGAGCTCTACGAGTGGACAAAGGTTGACATCACTGACGAGGCACAGAAGGAGCGCGCAAGCCAAATGATCGAGGATGCTGAGCCTTTTGAGGGAGAAGCTTTGCTCGATGCCAAGTGCTTCAAGTAAACTTGCTGTTTAATCTCTCTGATTGGCTTAGTTTCTTATTTGTTTTGTTGCTATGCTGAGTTATTTCTTTCTTTGCCTTTTTGACATAGCGTTGAGATTTTAGTACTCTGCATTTTATGGCTCCTTTTGAATGGTAGTCACTTTGTGACTTTATTTTGGGTTTTTGAGACTGCATCAGTAAAGGTACAATTTTGTATCAAGACCAATCTGTTATA

>*UBQ*

GTGAAAATCCTCTCTGCTTAATTACGTTCTACAATTCTTCCGTCGTTCCTTTATTATTCCCAGTTTCTTCTCTTATCGTCCTTTCTTTCTCTCTCTTATTTATTTTCTTCTCCGATTTCCCCGTATCTCTCGCTTCTTCTTGTTCTTTCCTTCTCACATCTTTTCTACACATTAAATATCGCAAATACTTTCGATTAATCGTATATATTGCTGTATAATTGAGCAACTCTAGGCTAGGGTTTTGTTTTTTCAATCGATCGGTCGGTTGATATTTTCGGGAGACAAGGATGGGTGGAGAGGGAGATTCGAGAGAAACGCTCGCCGCCGTCGGTGGAGAGGCGGCGGCGGCGGAGGTCGGGCAGGACTTGGCTACCGTGAACATTCGGTGCTCTAACGGCTCGAAGTTTTCGGTGCAGACGGCGCTGGGCTCGACCGTTGGTACGTTTAAGGTTCTGTTGGCTCAGAACTGCGATGTACCATCGGATCAACAGAGGCTGATTTACAAAGGCCGGATCTTGAAGGACGACCAGACCCTTGAGAGCTATGGTTTGCAAGCAGATCACACTGTTCACATGGTTCGTGGTTTTACTTCCTCTCCAACAACTACTGCACCTGGGAGTGGTACCACAAATGCCGGGAATGCCAACACTACTTCTAATGCTACACGTACTACTACCGCCCCCTTAAATGAAGGTGGTGGCTTAGGAGGTGCTGGTCTTGGCGCATCTCTTTTTCCTGGGCTTGGTTTGGGTGGCAATGCAGGATCTGGTTTATTTGGCGCTGGATTACCAGAATTGGAGCAGATGCAGCAACAGCTGACTCAAAATCCTAACATGATGAGAGAAATAATGAACATGCCTGCTATGCAGAGCCTGATGAATAATCCTGACTTGATTCGAAGCGTTATTATGAGCAATCCTCAGATGCGTGACATCATTGATCGTAACCCTGAACTTGCTCATATACTCAATGATCCAAGCATTCTTAGACAAACAATGGAGACTGCAAGAAACCCAGAGCTCATGCGTGAAATGATGCGCAACACTGATAGAGCTATGAGTAATATTGAATCCATGCCTGAAGGATTTAATATGCTTAGACGCATGTATGAGAATGTACAGGAACCCTTTCTCAATGCTACAACAATGTCTGGGGACGCTGGAACTAATTTAAGTTCAAATCCGTTTGCTGCTCTCTTGGGAAATCAAGGTGGTGGACAACAGCAGAGAGATGGATCTAACAACTCTTCTACAACTGACAATGATTCTAGTGCTGGTGCTGCTCCCAATACTAACCCCCTCCCAAACCCTTGGGGTCCTAGTGGGGGCACACAGACAAATGCTGCTCCGAGATCTCCTGCTGGAGATGTAAGGCCACCGGGATTAGGTGGTTTGGGGGGCTTAGGTGGTTTGGGTGGCTTAGCTGGTTTGGGTGGTTTAGGCAGTGGTGTCCCGGATCTGGAGCGCATGTTGTCAGGTGGTGGAGCTCCAGATCCAGCTGTTGTCAGTCAAATATTGCAGAATCCTGCCATTTCACAGATGATGCAAAGCCTCCTTTCAAACCCAGAATATATGAATCAGATTTTGAACACGAGTCCTCAACTTCGTGCTATGTCTGAATTGAATCCTCAGTTTCGTGAATTGATGCAAAATCCTGAGCTTCTTCGTATGTTAAGTTCACCTGAAATTATGCAGCAAATGCCAACTTTTCAGCAAACACTACTTTCTCAGCTGAACCAACCCCGTGCAAACCAGGATCCATCTCAGACTGGTGGAGCTGCAGGAAACCCAAACAATATGAATCTTGATATGTTAATGAGTATGCTCGGCGGACTGGGAACTGGCGGTGCTTTGGGCGTCCCTGAGACTCCAAATGTGCCTCCAGAAGAGCTATATGCGACACAACTTTCCCAGCTTCAGGAGATGGGATTCTTCGATGCACAGGCAAACATCAGGGCGTTACAGGCAACACGTGGGAATGTCCATGCAGCCGTTGAGTTCCTTCTGGGGAATCAGTGAGTTCCACCAAACTTATAAATTTCACATACTAAGAAAGAAGGGAGGAACACCACAAGAGCAAGGAACTTTGTTGTTACATCCTCGTCAGTTTTTTTTGGGGTTCCAAGGATTCCTGCTTTTAATTGAGCCAGGGAAACACAGCATGTAGTTGCCCAAGCCTGTAACTGGATTGGAACTTTGAGCTTGGGTAAATAAACAAAAGCAGGTAGACGAATGTCATAGAGTTATCAAGTCTGGTATACGCGTCTGTGTCTAAGGTCTCTCATTCCATTATCTTCTCTCGACTCTTTTGGAAACACG

>*UBC9*

TTTTCATGATTCTTCTTCTCCACCTCCCCCAATAAATTCGTCTTCATCCTCTCCTCTTCACACGCACACGCGGCTGGATCAGAGACCCAATCACAAGGGTAGCCATGTCAGGAGGCATTGCGCGCGGTCGTCTTATGGAGGAGAGGAAGGCATGGCGCAAGAATCATCCCCATGGTTTCGTTGCAAGGCCTGAGACTCTCCCTGATGGTACCATGAATTTGATGGCGTGGAGTTGCACCATTCCTGGCAAGGCTGGGACTGATTGGGAAGGCGGCTTCTTCCCCTTGACACTCCACTTCACGGAAGACTATCCTAGCAAGCCCCCAAAGTGTAAATTTCCTCCAGGATTCTTCCACCCCAATGTTTACCCCTCAGGAACTGTTTGCCTCTCCATCCTGAATGAAGACAGTGGATGGAGACCTGCGATTACTGTCAAGCAAATTCTTGTTGGGATCCAGGATCTGCTGGACCAGCCTAATCCGTTAGATCCTGCACAGACTGATGGATATCATATGTTTATCCAGGATAAAGTGGAGTATGGAAGGAGGGTTAGGCAGCAGGCAAAGCAATATCCTCCAGAAATTTGATGGTGTATCTATCTTGAAACTGCTGGAGAATGAACGTTGGTAGTTCATTTCAATTAAGTAATTGTGCAGGGTGCTGGTGTTTTCATGTGCTGACTCTTTAATGATAAAATATATATATAATATATAGCAGTAGAGTGATATAGGTCAGCTGATGGATGAAAAAGATTGGTGGTGTGTTCTTTTATTCGCTTTCTCTAGCTATCCGGTCAGTGATTGTATGAGAAATGTTTGCACTTGATGATTCTGATACTGGTTTGTGAAATGTTATCATAAAACAATTTGGATATCTAACTGCTTCTG

>*60SrRNA*

ATTATTAGGTTTTCAGTTTAGACTCGCCGTTCTCTCGGAGGCAGACAAAAAAAGCAGCAATGGCGAGAATCAAGGTTCATGAGCTGAGGCAGAAGTCGAAGGCCGATCTGCTGAGCCAGCTCAAGGAATTGAAGGCTGAGCTGGCTCTCCTTCGCGTTGCCAAGGTCACCGGTGGCGCTCCCAACAAGCTTTCCAAGATAAAAGTGGTGAGGTTGTCGATTGCGCAAGTGTTGACTGTGATTTCGCAGACGCAGAAGTCGGCATTGAGAGAGGCGTACATGAATAGGAAGTATTTGCCTCTTGATCTCCGCCCTAAGAAGACCAGAGCTATTCGCCGCCGTCTCACCAAGCACCAGGCCTCTCTGAAGACAGAAAGGGAAAAGAAGAGGGAGGTGTATTTCCCCTTGAGAAAGTATGCAATCAAGGTCTAAGATTGGAGTTTTTGATTAGTAGTGTGTTCAGTGCTTTACCAGTATTAATGCTGGATAAGAATGTTGATTTAGCTTTTGGTTTCATATTATTGGATATTTGTTTGAATACCTTCATCTTTGATCGAACTAGATTCAGTTCTATGGGATTGAGCTTAGTCCACAGAAATTCTGTATCTTTTGCTTCTTTTTTCTCAAGGAATACAAAATTTCATTTAAGAGACCCCCAC

>*eIF6A*

CCGGAGTTGCCACATTATAGAGAGGGAGAAGGGATTTGATTTCCAATTTCCAAACGCCGACTTCAATCGTGAGCCGCGACGCCCTTCTTTTTCGATACGCTTTGCTACTCACCCGGCCGCCCGTTCGGTGCCTTCACCGCCTCTCTATCCCTCTCCCTCTCCCTTCTTCGATTGATTCGTATTTGTGTGACTTTTGATTTGCTTGTTTAGCTCGTCATCATCTTCTTCTCCTCATGGCGACAAGGCTTCAATTTGAGAACAATTGTGAAGTCGGGGTTTTCTCCAAGCTGACTAACGCATATTGTCTGGTTGCAATTGGTGGATCCGAAAGCTTCTACAGCACATTTGAGTCTGAGCTGGCAGATTACATTCCCGTTGTGAAGACATCTGTTGGTGGAACTCGAATAATCGGACGGCTATGTGCTGGAAACAGGAAAGGACTTCTCTTGCCTCACACCACAACTGACCAGGAACTTCAACATTTGAGGAACAGTCTACCAGATGAAGTTGTTGTCCAGCGCATTGAGGAAAAACTGTCTGCTCTTGGAAACTGTATTGCTTGCAATGATTATGTTGCACTTACCCACACGGATCTTGACAGGGAAACCGAGGAGATGATTGCAGATGTTCTCGGTGTGGAAGTGTTTAGGCAGACAATTGCTGGCAATATCCTTGTAGGAAGCTACTGTGCTTTCTCCAACAAGGGAGGCCTGGTGCATCCTCACACATCTGTGGAAGACTTGGACGAACTCTCAACACTCCTTCAGGTGCCGTTGGTGGCTGGAACTGTGAACCGTGGAAGTGAAGTAATAGCTGCTGGGCTGACCGTAAACGACTGGACTGCCTTCTGTGGGTCCGACACCACAGCCACAGAACTCTCTGTTATTGAGAGCGTCTTCAAATTGAGGGAAGCTCAGCCAAGCACAATTGTTGATGAGATGAGGAAATCATTGATAGACACCTATGTCTAAACTCACCTGTGAAAACCTATGGTAATGTATTTTTTGGGTTTGTTTGTTCACTCTATGGACCATTTATGCTTTTTCAACTGTGCTCAATTTTGCGTGAATTTTTTAATCTTAGACGTATGGAACACCAGATTATTAATGCACTCGAGATTCTCTATAAATGTAACTCTTACAAGGGCGTACATGACAAATGCTTTTTGTTTTTTCTTCGTTTAGGACCAGCTTTGTGGTACAATGTCTTTTGCATGAGTGGGTCATACGAATATCAGAAAGTAACTGTGGCTTGGTTTTGCAACTCATGAT

>*SKD1*

GGACAATGCGCACGCTCCAGCTTTCTTCTACAATCTACCTTTGTCTTCTTTTCCCGTATTCTTCCCCCTTCCCAAATCTGAACTCCACGCGTCTAATTCACGCTCCTAGGGATTTTGGGATTTCTCTTCTTCCCTTCCCAAATCCCGATTCTCAGCAATTGGACGATCAATTTCAAATCGTTCATATGCGAATTCCCTAGCAACAGAACTTCTGAATCAAGAGAGGGGAAAACATGTACAGCAACTTCAAGGAGCAAGCGATCGAGTACGTCAAACAGGCTGTGCAAGAAGATAATGCCGGGAATTACGCCAAAGCTTTCCCTCTCTATATGAACGCTCTGGAGTATTTTAAGACGCATCTGAAGTACGAGAAAAACCCTAAGATCAAGGAAGCGATCACGCAGAAATTTACGGAATATCTTCGCCGCGCGGAGGAGATTAGGGCGGTGCTGGATGAGGGCGGTTCTGGCCCTGCCTCCAACGGAGACGCTGCGGTGGCAACGAAGCCGAAGACCAAGCCGAAGAACGGTGGCGATGGTGAGGGAGATGATCCGGAGAAGGAGAAGCTCAGGTCTGGATTGAACTCCGCGATCGTAAGGGAGAAGCCGAATGTGAAGTGGAATGATGTGGCTGGGTTGGAGAGTGCCAAGCAGGCGTTGCAGGAAGCGGTCATATTGCCTGTCAAGTTTCCTCAATTTTTTACTGGTAAAAGACGACCATGGAGAGCTTTTCTCTTGTATGGGCCACCTGGGACAGGAAAATCTTACCTAGCCAAGGCTGTTGCTACTGAAGCTGATTCGACATTCTTCAGGTAGTCATGGTTGTGTTATTCAAGTTTCTCTTGCAAAGACTTAGATGATGGTCTTTGGGTTTTAGTCAAGTAACATAGTCTTTGAAATAACTGTTGTCTGCAATACAAGTTATGCTGAACGATTGTCTATATAAGTGATCCATGTAGTTAATAGCTATAATTCTTCTAATTTGCCCATGTAAACATTGGATGGAATCATAAATCTCGACATGTACAAGTGCTACCACATAAACTATACAGAAGATTCGGGTCATATCATCAGCTGATGACAAAACCTTTGATATAAACTTCGAAAATATTCTCC

>*YLS8*

ATAAACCAAATTTAATTTTGGGAGAAAAAATAGGGTATAAATATGGGCAACTCGTGGCCTAATCCAGTAACGCGGGTCTTGCTTTACCAAATTGGAAGTTTGTTCTACGAAGAGAGGAGGAAGAGGATGTCGTACCTTCTGCCACACCTGCACTCCGGATGGGCGGTGGATCAGGCAATCCTCGCGGAGGAGGAACGCCTCGTCATCATCCGCTTCGGCCATGACTGGGACGAAACTTGCATGCAGATGGATGAAGTGCTGGCTTCAGTGGCAGAGACTATCAAGAACTTTGCTGTGATATACCTTGTGGACATAACAGAGGTGCCCGACTTCAACACAATGTACGAGCTGTACGACCCCTCAACCGTGATGTTCTTCTTTAGGAACAAGCACATTATGATTGATCTTGGAACTGGAAACAACAACAAGATCAACTGGGCTCTAAAGGACAAGCAGGAGTTTATTGACATTGTTGAGACGGTCTACCGTGGGGCCCGCAAGGGTCGAGGTCTGGTGATTGCACCTAAAGACTACTCCACTAAGTATCGCTACTGAGATCTCTATCTGGTGCTTTTATTCATAATAATTGGCCCGAGTTTGTGTTTTGGCTACTACTTGTGTTGTTGCTAGTAATTGGAAAAAGATTGAGATTCTGAACTTGTGATGATCTGTGAGTACGAATCTCCTTTCCTTTTTTTTTTTCCATTTTACCCCGCTTATATGGCTCTTATGAACCAAACAAGGCTTTGAGATGAAGCAAATCAAAGCTCAGCTTTCTACATCACATTTGGGAAAGAAGCCTCGCAAAAAAATTCAGGACCGTTGTGCATCCGCTTTCTCATTACAGAGCAAACA

>*NDUFA13*

TCATAACCTTCGCGTCCAGTTCCATCTCACGAAGCCCTTTACCTCAAACCAATTTTCATCGCCGCCGGCGACGACTTCCCCCTGAAATCCCCACACTTATCTCCGGAAAGCTGAACCTCCGCTTGAAGGACACTTAGAAGATGACGGAATCGATAATTAGGAACAAGCCAGGAATGGCGTCTGTCAAGGACATGCCCCTCCTCCAAGATGGGCCACCTCCGGGTGGGTTTGCTCCGGTCCGATACGCCCGTCGAATCCCAACCTCCGGTCCCAGTGCCATGGCCATCTTCTTGGCAACGTTCGGTGCTTTCTCTTGGGGCATGTACCAGGTCGGCGTAGGGAACAAGAAGCGCAGGGCACTTAAGGAGGAGAAATATGCTGCTAGGAGAGCTATACTACCTTTTCTGCAGGCAGAAGAGGATGAAAGGTTTGTGAAAGAGTGGAAAAAGTATTTGGAAGAGGAAGCCAGAATTATGAAGGATGTACCTGGTTGGAAAGTTGATGAGAGTGTTTACCACTCTGGTAGATGGATGCCTCCTGCCACAGGTGAACTTCGTCCTGAAGTCTGGTAAAACTAGAATAATTCCCTTTGCAAATCATGGATCATGATTCTTTCACCCAAGGGCTTCTTTGGTTTTCTTGTATGCAAGATTCTTCTGTTTGTTGCCCTTTTTAAGGATAGCAATAAATGTAACTGTGAATTTCTCAACGCTGTTAGCTGTTGACTATACTATGCTTTGCATCCATGCTCTTTAGCATTATGGGTAATGCGTTTTCACTTGATTGATAAATGATGACTGTTTAGCAATT

>*PcMYB4*

TCCCAACCCACACCACACCACATAAAATCTCTCCCTTTATTTCAACTCTCTCTCAAACCTTCTCTTCTTCCAACCTTCTCTTCTCTTTACTTTTCCCATAATTTCTTGCTTCAACAGTTAGCAGTTGATTATTTTTTTCTTGTGGAGTCATTAATTGTATACTCAATTATGGGGCGATCACCTTGTTGCGAGAAGGAGCACACCAACAAAGGAGCATGGACGAAAGAAGAAGACGACCGTCTTGTTAACTACATCAAATCTCATGGAGAAGGCTGTTGGCGTTCGCTCCCCAAAGCTGCTGGTCTTCAAAGATGCGGAAAGAGCTGCAGATTGAGATGGATAAACTACCTCAGACCTGATCTCAAGAGAGGAAATTTCACCGAGGAAGAAGATGATCTCATCATCAACCTTCACAGTTTACTAGGCAACAAATGGTCTCTTATAGCGGCTCGTCTTCCAGGACGAACCGATAATGAAATCAAGAATTACTGGAATACCCACATCAAAAGAAAGCTTATTACGCGCGGGATCGATCCTCAAACTCACCGTCCTTTTCATGCTTCTTCATCTCCAAACAACAAGAATGTTAGCAATGTAACAACCATGGCCACTTCAATTGATACCGCTACAACCACACAAACACAAAATGGAGGGTTCCAATTGATCAACATACAAAAAGGCTCTCTCTTGCAATTCGCTCCCACGCCCGAAACAAAAAACCCTAGTTTCACAAGACATGTGGCAAATTGTGCTCCCATTGTCGGAATCTCGGGCCGTGATGAAGATTCTAACAATAGCGTTGTGACGGGTGAAGATCAATTACTCGAAGGAGTCAACCTCGAGCTCTCTATTAGTCTTCCTTCTCCTCCGGCGAGGGTATCGCCTAGCAATGTCAAGCAAGAACAACAACAACAACAACAATATTCGTACTTGTGGAGAGGTTCAACCAACACATCAAGGCAATTGCCGGTGGCTCAACAAGGAGTTTGTTTGTGTTGCCATCTAGGGTTTCAAAACTCCAAATCTTCGTGTAATTGTTTGTCAATGACAACAACAATCACATATGGCCTATGAGCTTTTGTACGTGTTCATGGATCTCTTCTCTCTTCATCTTCCTTGCATTCGTGATCGAGTGTAATCCATTTAAGGAAATGAAGTTCATAGAAACATGAAATTCTCCAATGTAGCGGTCTTGTAAAATCTTCTGCTGGTTTCATAGTTGTTGATTAATTATTCAGTTTTAGTTCAC

>*PcPAL*

GGTGACCTTCTTCCCCAGGAAAATAATCCTTTTCATTTTCCTTCCCAAGTACGAAAGACCCAAAACAAAACAAAAAATAGGAAGCCTGTGATCTATTAAAAAAAACCAATTCAAGAAAAAGAAATGGAGGTTTCAAACGGGCATTGCAATGGAAACGGCGTCGCTTTGAACGGGCTATGCTTGAAGGAGGCGGCGAAGGTGAAGGCGTTAGCGGACCCGTTGAACTGGGGAGAGGCGGCCGAGGGGATGAAGGGGAGCCATTTGGACGACGTGAAGAGGATGGTGGAGGAGTTTAGGAAGCCGGTGGTGAAGTTGGGGGGAGAGACGCTGACGGTGGCGCAGGTGGCTGCGATTGCGGCGGCGGAAGAGGGTGGAGTGACGGTGGAGCTAGCGGAGGAGTCAAGGGCGGGGGTGAAGGCGAGTAGTGATTGGGTGATGGATAGCATGGACAAAGGGACGGATAGTTATGGGGTTACTACTGGATTTGGAGCCACGTCTCACCGCAGGACTAAGAACGGTGGTGCTCTCCAAAAGGAGCTTATTAGATTTCTGAACGCGGGAGTTTTCGGCAACGGGGTGGAATCATGCCACACCCTCCCCCACTCCACCACCAGGGCCGCCATGCTTGTACGGATCAACACCCTCCTCCAGGGCTACTCCGGCATCCGCTTCGAGATCCTCGAAACCCTCGCCAAGTTCCTCAACACCAACATCACCCCCTGCCTCCCTCTCCGCGGCACCATCACCGCTTCCGGTGACCTCGTCCCCCTCTCCTACATCGCCGGCCTCATCACCGGCCGGCCCAACTCGGTCGCCGTTGGCCCCGACGGCCGCCGCCTCTCCGCCTCCGAGGCATTCCAGCTCGCCGGCATCGAATCCGGCTTCTTTGAACTCCAGCCGAAGGAAGGCCTCGCCATGGTCAACGGCACCGCTGTCGGCTCCGGCCTCGCCTCCATGGTCCTCTTCGACGCCAACATCCTCGCGGTGTTCTCCGAGGTCCTCTCCGCTCTCTTCGCCGAGGTCATGAACGGAAAGCCAGAGTTCACCGACCACCTCACGCACAAGCTGAAGCATCACCCCGGCCAGATCGAGGCCGCTGCTATAATGGAGCACATTTTGGACGGATCTGGCTACGTGAAGCACGCGGAGAAGCTCCACGAATTGGACCCTCTCCAGAAGCCCAAACAGGACCGATACGCCCTTCGTACTTCTCCGCAGTGGCTCGGCCCTCAAATCGAAGTGATCCGAGCGGCTACCAAGATGATTGAGAGAGAAATCAACTCCGTCAACGACAATCCGTTGATCGATGTTTCCAGAAACAAGGCTCTGCACGGCGGAAACTTCCAGGGAACTCCGATTGGAGTTTCCATGGACAACACCAGGCTTGCCCTAGCCGCGATTGGAAAGCTGATGTTCGCTCAATTCTCCGAGCTCGTGAACGATTTCTACAACAACGGATTGCCGTCGAATCTCTCCGGCGGGAGAAACCCTAGCTTGGACTACGGCTTCAAGGGAGCGGAAATCGCGATGGCGTCTTACTGCTCAGAGCTGCAATTTCTAGCTAATCCGGTGACGAATCACGTCCAGAGCGCGGAGCAGCACAACCAGGACGTCAATTCCCTGGGCCTGATCTCGTCAAGAAAGACCGCTGAGGCGGTGGAGATTCTCCAGCTCATGTCCTCCACGTTCCTGGTGGCTCTGTGCCAAGCGATTGACCTCAGGCATCTGGAGGAGATCCTCAGGAACACCGTCAAGAACACAATCAGCCAAGTAGTGAAGCGAATATTGTCTGTCGGCGTCAACGGTGAGCTCCATCCGAGCAGATTCTGCGAGAAGGATCTTCTCAGAGTAGTCGACCGCGAGCACGTGTTCGCCTACATCGACGATCCTTGCAGCCCTAACTACGTCCTGATGCAGAACCTCAGACAAGTTCTGGTGGATCACGCCCTAGAAAACGGCGACAAGGAGAAGACCACCGCAACTTCGATCTTCCAGAAGATCGGTGCGTTCGAGGAGGAATTGAAGGTTGTGTTGCCAAAGGAGGTTGAAGGAGCAAGAAACGAGTATGAGAATGGAACTTCTGGAGTTGAGAACAGGATCAAGGAATGCAGATCGTTCCCGTTGTACAAGTTCGTGAGGGAGGAGCTCGGGACGAGCTTGTTGACTGGGGAGAAGGTTAGATCGCCTGGGGAGGATTTCGATAAGGTGTTCACTGCGATTTCTCGAGGATTGATGATTGATTCCTTGTTGGAATGCTTGAAGGAGTGGGATGGTGCTCCTCTGCCTATTTGCTAGTTTGTTTTTCATCTTTCTTCTTTTTTATTATGTTGTTGTTGTTGGTTTCTTGGACATCAATGGGTTGAATTTTTTTGTTGCTTTGTGACTCTTGTCTTGTTTAGATTCTATGCTAATTTATTTTCATAGTGTATGTAAGGCAAAAATAAGTGATTTGTTTGAAACTGGATTATGCAAGAATGAACCAGCTATATTTGATGATGTGGTAATAGTATGTACCATTTTGGCATCTGTTTGCTTTATGGTGTTATAAGAAGGGAAACAAACTAT

>*PcSTS*

GAAAACCCATTCGATCGATCATTAGTACTTCCAACTAAACTAGCAAGCTAGTGAGAGATGGCAGCTTCAACTGAAGAGATGACGAAGGCACTAACAGCCGCCACCGTCCTGGCCATCGGCACGGCCAATCCTCCCAATTGCTACTACCAAGCTGACTTTCCCGACTTCTACTTCCGCGCCACCAACAGCGACCACCTCACCCACCTCAAGCACAAATTCAAGCGCATTTGTGAGAAGTCAATGATCGAGAAGCGTTACCTTCAATTGACGGAAGACATTCTCAAAGAAAACCCGAATATCGGTGCGTACGAGGCACCATCATTGGATGTAAGACACGAAATTCAAGTGAAAGGAGTTGCACAGCTTGGGAAAGAGGCCGCTCTCAAGGCCATGCAAGAGTGGGGCCAACCCAAATCTAAGATCACACATCTCATCGTGTGTTGCATAGCCGGGGTTGACATGCCAGGCGCAGATTATCAACTCACTAAGCTTCTTGACCTAAACTCTTCTGTTAAGCGCTTCATGTTTTACCACCTAGGATGTTACGCTGGTGGCACCGTCCTTCGTCTTGCCAAGGATATAGCCGAGAACAACAAAGGAGCTCGTGTTCTCATAGTTTGTTCAGAGATGACGCCAATCTGCTTCCGTGGGCCATCTGAAACCCATATAGACTCCATGGTAGGGCAAGCAATATTTGGTGATGGTGCTGCAGCTGTCATAGTTGGAGCGAACCCAGACCTAACAGTTGAGAAGCCCATTTTCGAGTTGATTTCCACAGCCCAAACTATCATACCTGAATCTGATGGTGCGATTGAGGGCCATTTGCTAGAAGTTGGACTCAGTTTCCAACTCTACCAGAATGTCCCCGCACTAATCTCTAATAGCATAGGAACATGCCTTTCAGAAGCTTTCACCCCTCTAAACATTAGCAATTGGAACTCCCTCTTCTGGATCGCACATCCTGGTGGCCCTGCTATCCTAGACCATGTTGAGGCCACCGTTGGTCTCAACAAGGAGAAACTTAAGGCAACCAGACAAGTGCTGAACGACTATGGAAACATGTCAAGTGCTTGTGTGTTTTTTATCATGGATGAGATGAGGAAGAAGTCACTTGAAAACGGCCACGCAACGACTGGAGAAGGACTGCAGTGGGGCGTTCTGTTTGGATTCGGGCCTGGTATTACTGTTGAAACTGTGGTGCTACGAAGTGTGCCCATCATTTAACTCAAAATGATTGAATCACGTACTTCCAATTAGTAATATATGTTCATCCTTCTGTATCGGTAGAGTTGATCTGATGTCTTTGTGTTTTCTTTATTATTGAGCCCTTTTGATCATGTACCCTGAATAATTGTGAGTTTCCTCCACAATGGGAACGCTTGTATGATAGCATTTTCAATTTAAAGAATAACATTATATTCGATCTCC
