# Supplementary material for: Identification and evaluation of reference genes for quantitative real-time PCR analysis in Polygonum cuspidatum based on transcriptome data
Source: BMC Plant Biol. 2019 Nov 14;19:498. doi: 10.1186/s12870-019-2108-0 (PMC6854638; doi:10.1186/s12870-019-2108-0)
Supplement: Supplementary file 5 — Additional file 5. Genomic DNA sequences of 12 candidate reference genes and 3 target genes. The exons were shown in green shading, qPCR primers were marked yellow. [file 12870_2019_2108_MOESM5_ESM.docx]

>*ACT*

ATGGCTGACGAGGAAATCCAACCCCTTGTTTGTGACAATGGTACTGGTATGGTGAAGGTGAGTTCCATGCTGAATCTTGAGTTAATTTACTTAGTGTGAATCAGCAATATTGTTGTATGGGGAAAAAAAACTAATTGTTCAGTTTTTTTCTTTAAATAGGCTGGGTTTGCAGGAGATGATGCTCCTAGGGCTGTGTTCCCCAGTATTGTGGGTAGGCCAAGACACACTGGTGTTATGGTTGGCATGGGGCAGAAGGATGCTTACGTTGGTGACGAGGCTCAATCCAAAAGAGGTATTCTGACCTTGAAATACCCCATTGAGCACGGTATTGTCAGCAACTGGGATGACATGGAAAAGATCTGGCATCACACCTTCTACAACGAGCTTCGTGTTGCTCCTGAGGAACATCCAGTGCTTCTCACTGAGGCTCCCCTCAACCCTAAGGCCAACAGGGAGAAGATGACTCAAATCATGTTTGAGACATTCAATGTCCCTGCCATGTATGTTGCTATCCAGGCTGTTCTATCTCTCTATGCTAGTGGTCGTACAACTGGTTAGTATCTTTTCATCTCTTGATTCAATATTATGTATAATCCCATCATCTACATGTATTTACAGTGTATTATTCTTTTTTAACAGGTATTGTGCTGGATTCTGGTGATGGTGTGAGTCACACTGTCCCCATTTATGAAGGTTATGCTCTCCCCCACGCTATCCTTCGTCTGGACCTTGCTGGACGTGACCTTACTGATTCCCTCATGAAGATCCTTACTGAAAGAGGGTACATGTTCACCACCACTGCTGAACGGGAAATTGTCCGTGACATTAAGGAAAAGCTTGCGTATGTTGCTCTTGACTATGAGCAAGAGCTTGAAACCGCCAAGAGCAGCTCAGCCATTGAGAAGAACTATGAGCTTCCTGATGGACAAGTCATTACAATTGGTGCTGAGAGGTTCAGATGCCCTGAGGTTCTCTTCCAGCCGTCTATGATTGGAATGGAAGCTGCAGGAATTCACGAGACTACCTACAACTCCATCATGAAGTGTGATGTCGATATTAGGAAGGACTTGTACGGTAACATTGTGCTCAGTGGAGGTTCAACTATGTTCCCTGGTATAGCTGACAGGATGAGCAAGGAGATCACTGCCCTTGCTCCAAGCAGCATGAAGATCAAGGTTGTGGCTCCTCCTGAGAGGAAATACAGTGTCTGGATTGGAGGATCCATCCTTGCATCTCTCAGCACCTTCCAGCAGGTAGTTTTCTAGCTTCAATATTTTGAATATGGTTTCTTAAGGATATTCTTTTCAGGAAATATATGATTCTCCTGTACTATCATGACACATAATTCATAAATCATGTCCTTGAGTTCTGTAGAATGAACTAAATGTCATTTTCTGGTAATTTTATCAACATATGAACATGGTTGGAGAGTATTTTGGGGTGAGATAGTATATGGGTCATAGATTGCAAAGTGCGAGATAAGTAAGATAAAGAATATTAGGTAGATGAGAGCATATTATGAGGTTTCTATAATATTGGACAGAGGTCACTTTGTGAAGTACATACATCAGATTATGCCTTTGAAAGGGAATAAAGCAATGAATACAGGCTACAATATAGAATTTTAAAATTGTGTTTTCCAATTAATAAAGCAATGAATACAGGGTACAATATAGAATCTTAAAATTGTGTTTTCCAGCTAATAAAGGTTATAAAGCAACAAGTACAGGGTACTACAATATAGAATTTCCCTTCTATATCCTGCATCTGACATTATATTTCTGTATCGCTTCTCAGATGTGGATATCCAAGGGAGAATATGACGAATCCGGTCCAGCCATTGTCCACAGGAAGTGCTTCTAA

>*TUA*

ATGAGAGAGTGCATCTCGATCCACATTGGTCAGGCCGGTATCCAGGTCGGCAATGCCTGCTGGGAGCTTTACTGTCTCGAGCACGGTATCCAGGTATTTTCCTCTCCCTTTCTTTGACGCTGCGAAATTAGTTCAGATCTAGCGTTTATTATTGAATATTTTTACAGATCTGTAAGATTGACAATAGATATGTGAATTTGGCGTTCTTCCATTGTGTTGGGAAATTTGTGAATCTCAGTGTTACATATGATGGAGTCTGCGTATTTTGATACTTGAAAGGTCCAGATCTAGTTATATTCTTTGAAGGTATTTGCAGATCGGTATGATTAAAATATAGATCTGTGAAAGTATCTAGTTATTCTTTGAAGGTATTTGCAGATCGGTATGATTAAAATATAGATCTGTGAAGGTGTATGTGCATTTTGATAAATTTCTGCAATTACTTCAGATCTAGGGTATTTTCTTGAAGACAATTGCAGATCGGTAGGATTCCGCAGTAGATCTATGAATTTGATGTTTTTCCAATGTGTTGCGCGCCTTAGATCCGTAATGAGAGTAGTTGTTTGACATTTCTGTTGAAAAATCTCGTAATTACGGTAGATCTAGTTGATTGGGATTATCGACTTCATCCTTGAAAATGATGTGTTGGTCTCTAAATTGTATGACCTTTGTTTGTATCAGCCTGATGGCCAGATGCCAAGTGACAAAACTGTTGGTGGAGGTGATGATGCCTTCAACACCTTCTTCAGTGAGACTGGTGCTGGGAAGCACGTTCCTCGTGCTGTCTTTGTAGATCTTGAGCCTACTGTCATTGATGAAGTAAGGACTGGAACATACAGACAACTCTTCCACCCTGAGCAACTCATCAGTGGGAAAGAAGATGCTGCTAACAACTTTGCTCGTGGACACTACACAAGTAATTGCTCAATCCGATCTGCATTGCAGAATATCGAACATACTTTGTATCATTTTTTCTTGAAATCTCTTACAAATTCAATATTCTGATGTAAATGCTTTGGTGTTTCAGTTGGAAAGGAGATTGTGGATCTCTGCTTGGACAGGATCAGGAAGCTGGCTGACAACTGCACCGGTCTTCAAGGTTTCCTTGTATTCAATGCTGTTGGTGGTGGCACTGGATCTGGTCTGGGGTCACTTCTCCTAGAGAGGCTCTCAGTTGACTATGGAAAGAAGTCAAAGTTGGGTTTTACTGTCTACCCATCTCCACAAGTGTCAACCTCTGTTGTTGAGCCCTACAACAGTGTTCTATCCACCCACTCCCTCCTTGAGCACACTGATGTCTCTGTTCTCCTTGACAATGAAGCCATCTATGACATCTGCAGGCGCTCCCTGGACATTGAGCGCCCCACATACACCAACCTCAACCGTCTTGTCTCTCAGGTCATTTCTTCCCTGACAGCATCTCTGAGATTTGATGGAGCCCTGAATGTGGATGTGAATGAGTTCCAGACCAACCTTGTTCCCTACCCAAGGATCCACTTCATGCTTTCTTCCTATGCTCCAGTCATCTCTGCTGAGAAGGCCTACCATGAGCAGCTCTCAGTGGCTGAGATCACCAACAGTGCCTTTGAACCCTCATCCATGATGGCCAAGTGTGACCCACGTCACGGGAAGTACATGGCTTGCTGTCTGATGTACCGTGGTGATGTTGTGCCCAAGGATGTCAATGCTGCTGTTGCTACTATCAGGACTAAGCGCACCATCCAGTTTGTCGACTGGTGCCCTACTGGCTTCAAGTGTGGTATCAACTACCAGCCACCAACTGTGGTTCCTGGTGGTGACCTTGCCAAGGTTCAGAGGGCCGTTTGCATGATTTCCAACTCCACCAGTGTTGCTGAGGTGTTCTCTCGCATTGACCACAAGTTTGATCTGATGTATGCTAAGCGTGCCTTTGTTCACTGGTATGTGGGTGAGGGTATGGAGGAAGGAGAGTTCTCTGAAGCTCGTGAGGACCTTGCTGCTCTTGAGAAGGACTATGAAGAGGTTGGTGCTGAAGGTGTTGAGGGTGAGGATGATGATGGTGAAGAGTACTGA

>*TUB*

ATGCGTGAGATCCTTCACATCCAGGGTGGTCAGTGCGGGAACCAGATTGGGGCCAAGTTCTGGGAGGTGGTGTGCGCGGAGCACGGCATCGACTCCACTGGTCGCTACAATGGCGACTCAGAGCTTCAGCTTGAGAGGGTGAATGTGTACTACAACGAGGCCAGCTGTGGCCGCTTCGTCCCCAGGGCGGTGCTCATGGACTTGGAGCCTGGAACCATGGACAGTTTAAGATCTGGTCCCTACGGTCAGATCTTCAGGCCTGATAACTTCGTCTTTGGTCAGTCCGGTGCCGGCAATAACTGGGCTAAGGGGCACTATACCGAGGGTGCTGAGCTCATCGATTCTGTGCTTGATGTTGTGAGGAAGGAGGCCGAGAACTGTGATTGCTTGCAAGGTATAATTGTTGGTTTGATTCACATAGATTCAAAATTGTCCGTTTCTTATAGCTGATATCCAGTTACATGAAATTGTTTATGCTGATACTTTGAATTTGTTTTGTAGGATTCCAAGTTTGCCATTCACTTGGTGGAGGTACAGGTTCTGGTATGGGAACCCTTCTTATTTCGAAAATTCGGGAGGAGTATCCTGACCGGATGATGTTGACATTCTCTGTCTTCCCTTCACCGAAAGTATCCGACACTGTTGTTGAGCCTTACAATGCCACTTTATCAGTTCATCAGCTTGTTGAAAATGCTGATGAGTGTATGGTTTTGGACAATGAGGCTCTCTATGACATTTGCTTCCGGACTCTGAAGCTTTCAACCCCATCCTGTGGGTATTTGTTGTGTTTCTGTTTTCAATATATTCTACTACGATATAGATTTCCATCTTTGAATATTCTGTTTGTTGATGATGTTGAATACTGGGGCGATGATGTTGATGATGATGATGTTGAATATTCTGTTTGTTGTGGATAATTTTAATAACAAGAATGTTGCCACTCTTTCAACCCTCTAAATGAAATGTGTTTGTGTTTGTTTTTTTTTCTTGCAGTTGGGGATCTCAACCACCTCATCTCCGTCACCATGAGTGGTGTAACATGCTGTTTACGTTTCCCTGGCCAACTCAACTCCGACCTCCGTAAATTAGCAGTAAATCTCATCCCCTTCCCACGTCTCCACTTCTTCATGGTTGGCTTTGCCCCCTTAACCTCAAGAGGCTCCCAACAATACAGAGCTCTAACCGTCCCTGAACTCACCCAACAAATGTGGGACGCGAAAAACATGATGTGTGCAGCCGACCCACGCCATGGCAGGTACCTAACTGCTTCAGCTGTGTTCAGAGGCAAAATGAGCACAAAGGAAGTTGATGAGCAAATGATCAACATCCAGAACAAGAACTCGTCTTACTTTGTTGAGTGGATCCCGAATAATGTAAAGTCGAGCGTTTGTGATATTCCTCCAAGGGGATTGCAAATGTCTTCTACTTTCATTGGGAATTCGACGTCCATTCAAGAGATGTTTAGGAGAGTGAGCGAGCAGTTTACTGCCATGTTCAGGAGGAAGGCTTTCCTGCACTGGTACACGGGTGAAGGAATGGATGAGATGGAGTTTACTGAGGCGGAGAGCAATATGAATGATCTGGTGTCTGAGTATCAGCAGTACCAGGATGCAACTGCTGATGAGGAGGGTGAGTACGAGGATGAGGAGGAGGACGACGAGGAACAGTTTGCATGA

>*GAPDH*

ATGGCAAAGATCAAGATCGGAATCAACGGTAACGGTTCTCTTTTTCACGATCTTGTGCTTTTATGCTTCGATCTGGTCGTTTTACGCAAAAACGGCGTTGTACAATAAAATTAACGCAAAATCGATGTTTTTTGTTCAGGTTTCGGAAGAATCGGCCGTTTGGTCGCTAGGGTGATTCTTTCAAGGGAGGATGTGGAGCTCGTTGCCGTCAACGACCCCTTCATCACCACCGAATACATGGTAACTCCGATCTGCTAATATTTTAAGCTTTTCTTTAGATGCATGCTGTTAAGTTTTTGATTCGATGTTTGGTGAATTGGATAACGTAGATAATTGCCAGATCTTGTTATTTGTGCTGTTTAAGCGATCTACTTGACTTCTGGCTATAATTTTGGTTGTTCGCTTCTTGTTTATGGCTGGATCTTTCCCTGTTGTTTGTGCAGACATACATGTTCAAGTATGACACTGTCCACGGTCACTGGAAGCACCACGACGTCAAGGTCCAGGATGAGAAGACTCTTCTCTTTGGTCAGTCACCAGTGACTGTTTTCGGTTGCAGGTGAGCTTTTATAATAATTTGCGTTGAGTTTAATTGGGATGTCTTGTTATTGCTCTGTTGATAGATGTCATAACTATCTTTTTAATGGCATGTGAACTTCTGCTTATGATGTTACATTTCCCGTTACTAGGAACCCAGAGGAGATCCCATGGGCTCAGACTGGTGCTGACTTCGTTGTTGAGTCCACTGGAGTCTTCACCGACAAGGACAAGGCTGCTGCTCACTTGAAGGTTCGTGATTGTCATGTTTTGTACTAATTATTGATTTACTGTTTTGTGATTATGATTGTTCTTCTTGCATGCAAATACGGGCTGTGGATGTATTACAGCTTTTGTGGTTATACCCTACCCTATATCAATGTTTACCTGTAAATTATATTATTAATTAGTGATTGGTCACCTATTGATGAAGTAATCTTTCAGTAACTCGCTGTTATGTTGAATGTGCATGCCATGATGAGCAACTTGATTGCTTATTTCTTTACAGATCTTGTACTGTGTTATCTATGTAGTTAATTTGATTTGCTTATGTTATCTTGTTTCTAATTGGATCTCCCTAAATATATACTTAAGAGGGTACTTCTCACATGCTTTAGTTGTTTTTGCTTGATTTATTTTGATTTGATTTTAATTTAATTACATCTTTTATTTATGTTGATTAGTGGTAGTTTGTTTTTGACATATTAGGGTGGTGCTAAGAAGGTCGTCATCTCTGCACCTAGCAAGGATGCACCTATGTTTGTTGTTGGTGTTAATGAGCATGAGTACAAGCCTGAGCTTGACATTGTCTCCAATGCCAGTTGCACAACCAACTGCCTTGCTCCCTTGGCTAAGGTATGCTATCTGCTATGTTGGTGAGGTGTGTATGGAAAATATATGCGGGTGTTCCAAAATCTTATTCTACTTGTTTTAAATTATAGGTTATCAATGACAACTTTGGCATTGTTGAGGGCCTAATGACCACAGTTCACGCAATGACCGGTATGCCGCAAATTGTCTTTTTTATTACTACAGTCAGTTCTATACTAATTCAAAAGATTACAAAACTACATCCGGTTATGTTTTGATGTTGGTGTCTCCTTTTTCGTATTATAGCCACACAGAAGACTGTTGATGGTCCATCAATGAAGGACTGGAGAGGTGGAAGGGCTGCTTCATTCAACATCATTCCCAGCAGCACTGGAGCTGCTAAGGTCTAATTTACTTTTCTTTCAAGTACCGGTGGACGTATTTTATAAGCTGGATGTCACATTTCCTATCATTTTATCTGGTGCTATATGAATCAGGCTGTTGGCAAGGTGTTGCCTGCTTTGAATGGAAAGTTGACCGGAATGGCATTCCGTGTCCCAACTGTTGATGTGTCTGTGGTTGACCTCACTGTCAGACTTGAGAAGGCTGCCAGCTACGAGGACATTAAGCGTGCCATCAAGTACGTAAAATTGCACTCAGTGATCTTGTTTGTAGTTTGGAGTATAATTGCGTTGAATGGTCTTGTCCCGTGCAGGCTGATTAGTGTTCGTCTCGTTTTGCAGGGCGGCATCTGAGGGCAAGATGAAGGGAATTATGGGTTACACTGACGAGGATCTTGTTTCTACTGACTTTATCGGTGACAACAGGTAAGATTTGAACATCACTTCTGTACAGAGTTAAACCATTATTCCTCCCCACACTTTCCGTGAGTTTCTTCTTGGTTGAATTAGCTTTTGATTGATTTGGATACATGATTGCAGATCAAGCATCTTTGATGCCAAAGCTGGTATTGCTTTGAATGAGAATTTTGTCAAGCTTGTGTCCTGGTATGACAACGAAATGGGTTACAGGTAATTTGCCTCTCTACCCTTGTGTTTCTCCATCCTGCATCAGCTTCTCGCGGTTTTTTGTTATAATGCTGAGTTTTGTATTATGTCTGATGTGCAGTACCCGTGTCGTTGACTTGATCTGCCACATGGCCAAGTCCCTGTAA

>*EF-1γ*

ATGGCTCTGTTTTTGCACTCCGGAAGCAACAACAAGAATGCCTACAAGGCTCTCATTGCTGCTGAATACACTGGTGTTCGGGTTGAGTTAGCCAAGGATTTTAAAATGGGAGAATCCAACAAAACTCCAGATTTCCTTAAGATGAACCCTATCGGAAAGGTGAGATCTTTTTATGACCATCCCATTATTTTGTTGTTCTATCTCTGTTTTCTTAATTTTATTTGACCGTGCGCGGCTTTCCCAACTTAGGTTCCTGTACTGGAAACACCCGAGGGGCCTGTCTTTGAGAGCAATGCTATTGCACGCTATGGTGAGCTCTTTTCACCAACTCAAGTTGTAAATACAAAAGAAATAGAGTTTTCTATGTTTTCTGGATTTGTTGCATCTAAAGGAGCTTGAAATTTAGCTTCTCTTTGTTTTTGTTTTAATTGGCTAAAACCTCTACTTATTGATAAAGGCTAAATCCTGTTGGAATGCTGCCTCTCTCCTTATAGGATTAGTGGGAATTGGTCTTATTGTCTCAAAATTTGGGTTGAAGGAATGGGGCTTTTGCTCAATTGACCCTTTTATTCTGGAATTTAGAACTTACTTTTGCATGTTTACCATGTAGTGGCTCGCTTGAATGCCGACAGTACACTCTATGGGTCATCCCTGATTGATTACGTAAGTAATTTGTTGCTTTTTTATCTGACCAAGTTACAGCCCACTACTTATTGCCTTACTGATTCACTGATCTGTGATACTTTCTGAACTATTTTGTTTATATATCCTCTTTTGCAGGCTCATGTTGAGCAGTGGATGGATTTTGCATCCATGGAGATTGATGCAAATATTGCCAGGTGGCTATTTCCCCGTCTTGGCTTTATTGCCCACCTTCCCCTGGTCAGTAAAAGCTTCTATGTTGTTGCTTGTATAGTTGTATTATAATCTGTTAAACAAGTATTCTGCTAGCTGACAGTATGTTAATTTCTATTATATATCTTCAGGCTGAGGAATTTGCAATTTCTCAATTGAAGAGGTCTTTGGAAGCATTGAACACACATCTTGCTTCAAACACATTCCTGGTTGGACACGGTGTTACATTGGCTGACATTGTCATGACATGTAACTTGTATCTTGGGTTTAGCCGCATCATGACCAAAACTTTCACCAAGGATTTCCCACATGTTGAGAGATACTTCTGGACCATGGTTAACCAACCGAATTTCAAGAAGGTTTTGGGTGAAGTGAAACAAGCAACATCCGTTCCAGCAATTGAAAAGAAGCAGGCACAACCCGCCAAGCAGAAGCCTAAGGAAGAACCAAAGAAGGCAGCACCAAAACCCAAAGAAGAAGCTCCTGCTGAAGAGGAGGAGGAGGCTCCCAAGCCTAAACCAAAGAATCCTCTAGATCTTCTTCCTCCTAGTAAGATGATTCTAGATGAGTGGAAGAGGCTCTACTCTAACACTAAATCCAACTTCCGTGAGGTTGCTATCAAAGGTATAGAACTTGTTTGTTTCTTCTTCTTTTTTTCTACCTGATATAAGCATTAAATCCTCGTATTAGAGTTGTGCCCCTGTAGCAGAATTGGTTTATTTATTTAATAGTCTTTCTAACTAAGGGCATAGATTAAGAATGCCATATTTAGAAACTAAACCCTAAAAAGGAAATCATAAACCCTATACAAGTAATTATATAATTTTAAACAAATAAATTTAATCAGAATCTTTTCAAATAATGGGTCTTAATAGGTTAGAAAAACTGTTCATTTAATTGCATTGAAAAGTATCATTCCAGAAACTCAAATCAATTGTATTTTTAATGACTGTTAAGCAAAACAGATTCCTAATAATTCTGCGATTGGAGATTAAACTGTGTTTTTGATAGAAGGCTTGAAGTTTAATCCGTGTCACTTTTTCTATGAAATCAGGATTCTGGGATATGTATGATCCTGAAGGATACTCTTTGTGGTTCTGTAACTACAAGTACAATGAGGAGAACACCGTCTCATTTGTTACTCTAAACAAAGTTGGTGGATTCCTGCAAAGAATGGATTTGGCGCGCAAGTATGCCTTTGGAAAGATGTTGGTGATTGGCTCTGAACCTCCATTTAAGGTCAAGGGATTGTGGCTATTCCGTGGACAAGAAATTCCCAAGTTTGTGATGGATGAGTGCTATGACATGGAGCTCTACGAGTGGACAAAGGTTGACATCACTGACGAGGCACAGAAGGAGCGCGCAAGCCAAATGATCGAGGATGCTGAGCCTTTTGAGGGAGAAGCTTTGCTCGATGCCAAGTGCTTCAAGTAA

>*UBQ*

ATGGGTGGAGAGGGAGATTCGAGAGAAACGCTCGCCGCCGTCGGTGGAGAGGCGGCGGCGGCGGAGGTCGGGCAGGACTTGGCTACCGTGAACATTCGGTGCTCTAACGGCTCGAAGTTTTCGGTGCAGACGGCGCTGGGCTCGACCGTTGGTACGTTTAAGGTTCTGTTGGCTCAGAACTGCGATGTACCATCGGATCAACAGAGGCTGATTTACAAAGGCCGGATCTTGAAGGACGACCAGACCCTTGAGAGCTATGGTATGCGATCTGGTCGATGTTGCGGTTTCAATTTATGTATTTTCCGATTGCTTTGGGATTTGAGCGATTTTAGGGTGGATGAATTCTTGAATTTTGGGCTCAATTAGCGGATTTTGGTCGATTGTTGCGAAAGTAATGTGATATTGATGTCTCTTTTAGATTTGGTTTTATCTTTGTGGATTTCGTGTCTGAATGAGAAGGGTTATTAATTGGTAGATGTGATTTCGTGTCTGACTTTGATCTAAATATTTGGGTTATTGAATCATGTTGAGCTGCTGTTTTCCTTCATCCTGACCAATATGTTTGGCTACATTAGCTTCATCTTTATGGATTGTTGTTTGTTCCTTGTTGATTGACTCCTTCTCTCTACTATCTAGTTGCTACATTTGGCAGATTCATCTTTGCAGCTTTGTGGCGTCGTTTTTTTTATCTTGGTGTTTCTACTTCTGGTTATGCCGTCTCACAAACCTTTTGGCTACATTTGTCAGCTTAGTTTTTGCGGCTTTGTCTATTGTGGTTGATGTCTTTCTGTCTCTCTACACTAATACTGGCTAAATCTGGCAGCTTTATCGACACAGCTATCATTTCCTGTCTCCAGTTCTGGTTATGAATTGAGTTGCAGTTATCCATCCTCACCTACCTTTTTGCTACATCTGGAACTGGAACCCTCATATGGCCTTTTTGGTTGATCGGTTTCTGTGTCAATAATTCGGGTTCTAGTAACCTGTAGAGCCTGACCTAAATTTTAATGTAATTGCTACCTAGAAGTGCTAAAAGATGACTGTAATCTGTTTGCTGATCAATAACTTGTCCGTTTTTTAATGTCAGCATGTGCAGTTTAAAATTTTTGTTAGTTGACCAATTAGCCTTGCTAGTTTTCTTTGAAGAATCTTCAATGTTTCTTTATTTGTCTTTTATGATGGTATTTCCTTTTTGTCATTATTTCAACAGTCACACATTGTGTGTTTTACTATTCCAATTAATTTGGAGAAACTCACTTTGTTGAGGGAATGGATTATTATACTGTGGTTATCAAAAAGAAACATTTATGCAATGCTATTGCATGAATAATTGGAAAATTCATCTTTAGTCTTTGCTTAAGACTTCACTGCATAGTATCCTGGGTATTTTTTTTTTAAACTATCAGGAGTCCATAAATTTTGTTCTGAAGATAACAACTAAAATGCAATATGAGTTAATGGTGTAGGAATTTTGCTTTTTTTTGGTGTGTGTTTGATCACCCTCGTTTTTTTGGGCATTGCATTGTGATTGGTGGATCAGAACTTCGATATATACTCAAACAATTTATCATCTTGTTATTTGTTATTCAAAGAATCTGATGTGGTTTCTGTGACTTCTCTTTCCCAGTTGGACTAAATGTGATCACTTCATATTATTTTGTCATCTGATTTGCTTATTTGCCTTGTAGTATTAAGTATTAACACTTTTTTGAGATATTTTTTCAAATTTCATTGTAAAATAACATTTCTTTGATGCTCCTTCCTGTCATATGCTGAATCCTGGATGACTGTTATAACTTAAATCTTTTTTTTGAAGTGTAAACTCTTCCCTTCTTGTTTCTGAAGGTTTGCAAGCAGATCACACTGTTCACATGGTTCGTGGTTTTACTTCCTCTCCAACAACTACTGCACCTGGGAGTGGTACCACAAATGCCGGGAATGCCAACACTACTTCTAATGCTACACGTACTACTACCGCCCCCTTAAATGAAGGTGGTGGCTTAGGAGGTGCTGGTCTTGGCGCATCTCTTTTTCCTGGGCTTGGTTTGGGTGGCAATGCAGGATCTGGTTTATTTGGCGCTGGATTACCAGAATTGGAGCAGATGCAGCAACAGCTGACTCAAAATCCTAACATGATGAGAGAAATAATGAACATGCCTGCTATGCAGAGCCTGATGAATAATCCTGACTTGATTCGAAGCGTTATTATGAGCAATCCTCAGATGCGTGACATCATTGATCGTAACCCTGAACTTGCTCATATACTCAATGATCCAAGCATTCTTAGACAAACAATGGAGACTGCAAGAAACCCAGAGCTCATGCGTGAAATGATGCGCAACACTGATAGAGCTATGAGTAATATTGAATCCATGCCTGAAGGATTTAATATGCTTAGACGCATGTATGAGAATGTACAGGAACCCTTTCTCAATGCTACAACAATGTCTGGGGACGCTGGAACTAATTTAAGTTCAAATCCGTTTGCTGCTCTCTTGGGAAATCAAGGTGGTGGACAACAGCAGAGAGATGGATCTAACAACTCTTCTACAACTGACAATGATTCTAGTGCTGGTGCTGCTCCCAATACTAACCCCCTCCCAAACCCTTGGGGTCCTAGTGGTAAGTTTGATATCTCTGTTTTTTTTTCTTGCTTCACTTTTCCTACTTCATGCAAATGGCTTCTGTTGCTGTTCAATTCTTTCCTAGTTTCTGAAATGCAGTCTCCTCTTACCAAAGTTGGCGACAAATTGTTAAATATATCTTTCTTGTTTTGGACTGTGACCTTCAGTTTTTCTTACACGTAATAATAATTATCTTCACCTTGCAACTGTATCCTTTTTACTTTGTGAATATCAGGGGGCACACAGACAAATGCTGCTCCGAGATCTCCTGCTGGAGATGTAAGGCCACCGGGATTAGGTGGTTTGGGGGGCTTAGGTGGTTTGGGTGGCTTAGCTGGTTTGGGTGGTTTAGGCAGTGGTGTCCCGGATCTGGAGCGCATGTTGTCAGGTGGTGGAGCTCCAGATCCAGCTGTTGTCAGTCAAATATTGCAGAATCCTGCCATTTCACAGATGATGCAAAGCCTCCTTTCAAACCCAGAATATATGAATCAGGTAATTTTGGCAAGCATTTATATAGAGAATGAGATGCGTATGAACTTGAATTGGATTCTTAGTCATGTACTTTAATCTTGTAGATTTTGAACACGAGTCCTCAACTTCGTGCTATGTCTGAATTGAATCCTCAGTTTCGTGAATTGATGCAAAATCCTGAGCTTCTTCGTATGTTAAGTTCACCTGAAATTATGCAGGTATAGTTCAAATGCTTTAGTTTGTTATTTTCATGCTGTCTCTTTCCATCGGGATGAGATGAATGCACTTCAAGAATGGAGAGGAATTCCTATTCGTTGCCTTTTAGATTTTTGTTTTGTTGTGTCTTAAAAGTCTAATCACTTTTATTTTTATTACTCTAATGTTCTCTCTTTCCCTAATATTACATTTGTGTCCTCTCTCTTTGCAGCAAATGCCAACTTTTCAGCAAACACTACTTTCTCAGCTGAACCAACCCCGTGCAAACCAGTAAGTTTGAATTGGCTAGATAATTTTATTTACCGACCAGAAAATATTGTGAATGTTTTTCTTATGCATTGCACATGCTTTTTCTTTCCGATTTTCATTTTGTATTAAAGTTGGTTTCCTTGATCAATGTTTCCCACAGCTTGTCAGGGCTAGAACTGATATTATCCTTGAAATTTTTACAACTATGTCCTCCTTAATTGCTGAGCCCCATTAGCTTCCGATATTTCTCTTTATTTTGCGTTTAAGAAGTAAGAACCACATAGTCAGTGGTTACTGGTTGGTCTTTGAGATTTTATATCTTCCTGCTGATGGTGTTACCATAGATTTGTACTTGTTGTTTTCTCTTCTTGGACTTGGCAAAATTTCGTTATGTGTGTACTTAACATTAAAAATTGCATTACAGGGATCCATCTCAGACTGGTGGAGCTGCAGGTATCCTTTCTGTTTTAGCATCCTTCTTTTTATATATATCTTCTTATCATGATGTCTAGTTTATGAGACTTCTATCTACAAACTACAGGAAACCCAAACAATATGAATCTTGATATGTTAATGAGTATGCTCGGCGGACTGGGAACTGGCGGTGCTTTGGGCGTCCCTGAGACTCCAAATGGTGACAACAACTCCTTGAATTTCCCTATACATATTTTTTTTTTCTCCATGCTGATACATCAACATTTTTTGGTTTTTGTTTTAAAAAATAGTGCCTCCAGAAGAGCTATATGCGACACAACTTTCCCAGCTTCAGGAGATGGGATTCTTCGATGCACAGGCAAACATCAGGGCGTTACAGGCAACACGTGGGAATGTCCATGCAGCCGTTGAGTTCCTTCTGGGGAATCAGTGA

>*UBC9*

ATGTCAGGAGGCATTGCGCGCGGTCGTCTTATGGAGGAGAGGAAGGCATGGCGCAAGAATCATCCCCATGTTCGACTTCTCCCCCCCAATTTCCCCCTTTCTAATCTCGATTTACGTTGTTTATTCGAGTGATTTCTGATTTCGGCTTTGTTTTCTGTTTGATTTGTGGCGGAATTCAGGGTTTCGTTGCAAGGCCTGAGACTCTCCCTGATGGTACCATGAATTTGATGGCGTGGAGTTGCACCATTCCTGGCAAGGCTGGGGTTAGTCTCTCTGTCTTTTATGCTTCAACACTTGTGTGCCTGTGAATCAGTGATTTCAATTGAAAAATCCGTGTCGGATAAGAGTATCGGCGGCTTGATTGTGTTTCGATTTTGATCTTCTATTGATTATTGCGTTTTTTGAATTGCTATCGTGGTGATTTTTGGGCCGTACAGTCTTTTTCTGTAGCTTTTACTAGATTAAGCGATTGATGATGTGATATCTGCAATTGTAATAATGAGTAAACTTCTCCACTTTGAAGGGACAGTATAGTTAATTGGTCATTTTCCCTGTTTATGGCTTTACTTTATAAGCTCCCCCGCTGATTGATATTGATTTCGACGTATAAATTGCATGTTTTAATCTCGGTTCTCATGATGATTAAGCTCATTGGTTCTCAGATTTCCTCTGATGCATCACCAAAGTCAAGTTTGTAAGGTCTTTATAGCTATTCATTAGTAAGAACACAGAGTATATAGTGTCATAAGAGTTTGGTGCTATTATTGACATACTGTAATTGCTGCATTTCTAGACTGATTGGGAAGGCGGCTTCTTCCCCTTGACACTCCACTTCACGGAAGACTATCCTAGCAAGCCCCCAAAGTGTAAATTTCCTCCAGGATTCTTCCACCCCAATGTTTACCCCTCAGGAACTGTTTGCCTCTCCATCCTGAATGAAGACAGTGTAAGTAGCTGTGCAATTGTGGACTTGTTACTAGATCCTAGTTATCTTTAGCTTGCATTTCTGATATGGAGGTGCGTTTTCTTCTTTGTTCTGCCTTTTTTTCAGGGATGGAGACCTGCGATTACTGTCAAGCAAATTCTTGTTGGGATCCAGGATCTGCTGGACCAGCCTAATCCGTTAGATCCTGCACAGACTGATGGATATCATATGTTTATCCAGGTACCCTTGTAACTTGTTTGATCTGATCAATCTGAATTCACAAACATTTTGGACTATATCTGTTTGATCATAGAATTTGAGTCACTCATCTCCCATTTAACATGAGTACTAGTATAATGGATTGCTAATGTTCAGTATTGAATTCTCTTCGAAAGAAAAGAAAACAGAGAGGATTCTGGAATATGTCTGTTTGAATCTTTGATCATAGACTGATGGATATCATATGTTATCAGGCTAAACATTATGTAAGCCAGAAAATAGAGATAAATGAAAGGAATCAAAATGTTCGTTTGTTTTTTTTTTTTTATCTGTACTATGAGCTAATCCAAATTGCTTATATAATGCAGGATAAAGTGGAGTATGGAAGGAGGGTTAGGCAGCAGGCAAAGCAATATCCTCCAGAAATTTGA

>*60SrRNA*

ATGGCGAGAATCAAGGTTCATGAGCTGAGGCAGAAGTCGAAGGCCGATCTGCTGAGCCAGCTCAAGGAATTGAAGGCTGAGCTGGCTCTCCTTCGCGTTGCCAAGGTCACCGGTGGCGCTCCCAACAAGCTTTCCAAGATGTACGTCTCAATTTGATTTTTAGGGTTTTGTTTTTCGATTGTCTGGTCTAGTTGTTCGTTTTCTAATTGTGCTGGATTTCAATTTGTGTGGCTGATGCAGAAAAGTGGTGAGGTTGTCGATTGCGCAAGTGTTGACTGTGATTTCGCAGACGCAGAAGTCGGCATTGAGAGAGGCGTACATGAATAGGAAGTATTTGCCTCTTGATCTCCGCCCTAAGAAGACCAGAGCTATTCGCCGCCGTCTCACCAAGCACCAGGTATTGTAACCGATCTCATTCTCTCAATCACCATTTCTGGTAATTGATATAGTAAAATGGATTTGGTAACTATGTATGGACTAGTTTTCGTGATTGATTCTTGATTTCTGAGGTTTTATGTATGGTTGATTGCCTTTTGCTTGTTAAATTAGCGTTTAGTTCAAAGAATGGTTGTTATTTTAGTCCCAATTGGTGTCTGTGACAAGATTCATCATTGGATACATGCAATATAACTGAACAAGTGAACAAAGATTGAACTTTGATGAAATGACAATAGAACTATATAGCTAAGACTACAGATAGAAATTTGAATTTGACATTAATGAACTGAGGACTTATCTGTATCTGAATGTGACTTGTCTGCTTATGAAATTTACGTACTTGGTTTTTTTCCAGGCCTCTCTGAAGACAGAAAGGGAAAAGAAGAGGGAGGTGTATTTCCCCTTGAGAAAGTATGCAATCAAGGTCTAA

>*eIF6A*

ATGGCGACAAGGCTTCAATTTGAGAACAATTGTGAAGTCGGGGTTTTCTCCAAGCTGACTAACGCATATTGTCTGGTTGCAATTGGTGGATCCGAAAGCTTCTACAGGTTCAGCTTTCTTGTTGTGATTTTTATTTTCTTAAGAACTTGTTGACATGGGACCTTTGGTTTTAATTTGTTTTCGCTGGCGATGTCTATAGCACATTTGAGTCTGAGCTGGCAGATTACATTCCCGTTGTGAAGACATCTGTTGGTGGAACTCGAATAATCGGACGGCTATGTGCTGGTTAGCTCTGGCTTTTTCAAGAAAGCATAAACACTTTCTTTTTATTTGTAGTAAGTGCATTTATTCACATGCTTGCCTTCTTTCTGTTGGCAGGAAACAGGAAAGGACTTCTCTTGCCTCACACCACAACTGACCAGGGTGAGTCTTATACATGGGCAGACAACCGCTGTGCTATTCTTATGAGAACTTAATTTACTTATTATTTCTCATGGATTGGAGGTTGAATCGCCTCTGATTAATCTGTCATGCGTCATGATGTTTTAATGTGGAAATAATTTCGTGCAACAAAAACAATATAGTTGGCTTACTAGCTTTAGTGTTTGATTTTGCTTTCTGTCAAATTTTGTCTTTCTAAGAGCTTTTGTTGTTTGAGTGTTTTGATTGTGTTAAACTGAAATTGGAATTCAATATTTTGTGGTTTAATCTACCTTCAGTGCTGTGTTGCAGCTTCTGCAACATGAATGTTTTGTATTGTTTTCTAGAAATTCTCCATAAGAAGTGCATATTTTTAACTTGTGTGAGCTATAAACATGCTTCTGGAAGAATCATGTATAACGAACAAGAATCTCGAGGCATCATCATGACTAACAACTTCTGTGGCCCTTTTATGTTTGTCTCGAGTCTTCAGTAACATGTTCCTTTTAACCTGTTCCTTTTAACATGTTCTTACTGTGTGTCTTCTGTTCTTGTAGAACTTCAACATTTGAGGAACAGTCTACCAGATGAAGTTGTTGTCCAGCGCATTGAGGAAAAACTGTCTGCTCTTGGAAACTGTATTGCTTGCAATGATTATGTTGCACTTACCCACACGGATCTTGACAGGGTAACTTCCACATACTATATGTTTTTTTTTAATTTGCAGTTAATTTTATTAATGCTGGTCTTGGTGAATAATTCACTGTTTCTGGTATTCTTTTGACTGCGGTGGTTGATGTTCTTTATCTGGATGTCATGATACCGTGTCAATGTTGTAATTTGAGCGTTTCGACTTATTAAAGTATGTTTGCTAGAATGGGATCCGGCATATCGTGCTCTTTTTTCCCCCAAGTTTATAAGAAATGTTATATAGAAGTTTATGGGAAGCTAAAACTGGACAAGCTACTTTTCCTTACTCCATCCTTGTTTCCTGAAACTGTCAGTTCGCCATGTAAGATTGTAGTAGGGCAATAGAACTTTAGAAGTCGCTTTGTTTGCTGTTATAGATGCACGTAGACTTGAGTTGTCCCTTGTGTCTTTCTGTATTGATATTTTTGAGACCTTGTGGGTTTACAGAGTGGGCGGAGTGCTGGAGTTTGGATTTATCATTCCTGTCAATCATCTTATCTCTATTGAATGGAGTTACGAGATACCTATAAACAAGTTTTGATGTGCTGAGTTTCCTGCTCATTTATCATCATCTTCGAATTCAATAGCAGGCGTCAATGTTAATTGTTATTATTGTTTTGATTCTTTTCAATGATATGCCACCAGGAAACCGAGGAGATGATTGCAGATGTTCTCGGTGTGGAAGTGTTTAGGCAGACAATTGCTGGCAATATCCTTGTAGGAAGCTACTGTGCTTTCTCCAACAAGGGAGGCCTGGTGAGTACAACACTATCATTTTCTCCAATATCCTTTCCTTAATATTAAAAAGAAAAACCTTTCATATACGAGCTTTCAACTATTTGACATTGTCGATGATAGGTGCATCCTCACACATCTGTGGAAGACTTGGACGAACTCTCAACACTCCTTCAGGTGCCGTTGGTGGCTGGAACTGTGAACCGTGGAAGTGAAGTAATAGCTGCTGGGCTGACCGTAAACGACTGGACTGCCTTCTGTGGGTCCGACACCACAGCCACAGAACTCTCTGTTATTGAGAGCGTCTTCAAATTGAGGGAAGCTCAGCCAAGCACAATTGTTGATGAGATGAGGAAATCATTGATAGACACCTATGTCTAA

>*SKD1*

ATGTACAGCAACTTCAAGGAGCAAGCGATCGAGTACGTCAAACAGGCTGTGCAAGAAGATAATGCCGGGAATTACGCCAAAGCTTTCCCTCTCTATATGAACGCTCTGGAGTATTTTAAGACGCATCTGAAGTACGAGAAAAACCCTAAGATCAAGGAAGCGATCACGCAGAAATTTACGGAATATCTTCGCCGCGCGGAGGAGATTAGGGCGGTGCTGGATGAGGGCGGTTCTGGCCCTGCCTCCAACGGAGACGCTGCGGTGGCAACGAAGCCGAAGACCAAGCCGAAGAACGGTGGCGATGGTGAGGGAGATGATCCGGAGAAGGAGAAGCTCAGGTCTGGATTGAACTCCGCGATCGTAAGGGAGAAGCCGAATGTGAAGTGGAATGATGTGGCTGGGTTGGAGAGTGCCAAGCAGGCGTTGCAGGAAGCGGTCATATTGCCTGTCAAGTTTCCTCAATTTTTTACTGGTGAGTTGAGGGAATGCTTGAGTTTTTCCTGTGATTTTCAGATTAGGTTTAATTGGATGCTTCTTACTGGATTTAGGAATGCTTAAGTAGGATTCTTGTTCCTTTCCACACACACAAAAAAAAAAGAGAAAAAAGTAGGATGCTTGCATCGAACTTGCTTTCAGAGATTGTTTCTTAGAGATTGTTGGGAGTATCTGATGTTTAGTCTGTAGACTGGTATTGTTGGATGATGATCATGATAAGGGGCTCTACTGTATCTGACTATTTTGTTGCTGGATTCCATGCTTAAAACTATATAATTCATAGGATTGAGCTTTGTCTGATTAATGATTATGCATTGTTATCATTCTAGTGAATCAAAGGTCTTGAAAATATGTTGCTATGCTAATAGAAGATTTACATTGGCAAAGCAAGTCACTTGGTTTGTGTTAAGTAGGCAGAAATCTTGTCAGCTTTTGCTGAATATAAAAGAGCCTGCAAGTTAGTTTCTAGTTGCTTATTACTTCTAGGCATTAAATTTACGTTGAAGCAAAACTTTCTGTATATATTTTTGTATGAGAATTGCCTGATAATACCGTGTATGCCTTTAATTTAGGTAAAAGACGACCATGGAGAGCTTTTCTCTTGTATGGGCCACCTGGGACAGGAAAATCTTACCTAGCCAAGGCTGTTGCTACTGAAGCTGATTCGACATTCTTCAGGTAG

>*YLS8*

ATGGGCAACTCGTGGCCTAATCCAGTAACGCGGGTCTTGCTTTACCAAATTGGAAGTTTGTTCTACGAAGAGAGGAGGAAGAGGATGTCGTACCTTCTGCCACACCTGCACTCCGGATGGGCGGTGGATCAGGCAATCCTCGCGGAGGAGGAACGCCTCGTCATCATCCGCTTCGGCCATGACTGGGACGAAACTTGCATGCAGGTCCTCTCTTCCTTCCCTTCTCCTTTAAAACTCATACTCAATTTTCATTCCGGTTTTTTTTGCATTTTGTTTTTTGATCAAGTGCGTCGTGTGTTGGATTTTGGGACCCTGGCGATGCTTTCTTTCAAAGCCCTAATCCTAGATTTGTTAATTGTCGATTCTTGTATTAGCGGTTTACCTATTGCAAAACCCTAATTTTGTTTTAACTATTGTTTGCTTTTGGATTCTTGCTTGATGCGATGGTGATCTAGGGTTACCTTGATCTGCAGCTATAATCAGCAGTTTCTGTATTTTTTTTGCAGGATTTAGTTTGGGATTTGCTAGTAATTTAAGCAAGTTAGAAAGCAAGGCCGAATATAGGAGATCTTAACTAAATTATCTTCAACTTGGAGACGGATTTTAAAATTTATTTTTTTCCAATCCTTTTGCTTTTTGTTAAACATATTGATTTCCTGACATGTATGTTATCTTAACAATGGAAGAAATTCTTTTTATGTCTACCCTCGTATAGAATATTGAAAGCTAGCTTCCTCTTTTCAAGTCTCTTGACACCCCCTGTTAGCAGGATGTCCTATGAAATTCTAGGCTATGAATATAGTAATCCCTAGACATCATCTTATTACACATGTTTTGTAGTTTGCTAAAAACCAGTGCATTGTCAAAGATATGGCATGTTTCCTTCTAGTTGCACTTGTTCAACTCTTGTAGTAGAATAACAATTGTACCATTGTTCATTCACTTGATCTGTGACTCTGCTGATTACTTGTCTGAGTTGTCTTCTTGTAGGACTTCTGTAATTTTCAGATTATCCTATGTAAGCATTGCATAAACGTGGTAGATTTGTTATAATTGATGCTTTGATCAGTTTTTATACCCTCAGCCTCACCTGATTGTCCTGGTACTGAGATATGTTAGTATATGGCTAGAAGTTCTGAATTCCTTACAATCAGGCTTAGGTCTTTTCCCAAGCATTTATATGTTAGTAACATGGGCTGGTTGCAGGCATTCCTTATCACCAGCAAGGCTACACAACTAAGCTAAGACCTTCTATGATGACACTGAACTACAAAAAGGGACATTTTATAAAAGCTACAATAATGCTGTTAGAAGAAACCTGGTAATAGCTTGTTTTAAAACTCTTGTTTTGCTAGACCCAATTCCTTAGGTTCGAAGTTCAGAAGCTACAATAATGCTATTAAAAGGAAAATAGTCATAACTTGATTTAAATAGTTTCCTTTGTCCTAACCAACACTTGTATGTTCAGTCATTCGATTGCCTTGTAAAATTGAGTAAAATATAGCTAATATTCAAGTTGGAGTTCATACTTAATATAGTATAGTCATCCTTCTATCATCCCCTTCACCTCTGTCTTGTTAAATGTATGAATTTCTTTTGCATCACTGGTAACATCTTATCCATGTTTTGTTTGCTAATTGAAGTGATTATCCTCCAAAATCTGGGAAATATTGACTTGTACAGTACTTTTGATACACCTTTATAATTTATTTCTTTTCTTTCAAGTAATTTCTGAAATACAATAACTGACTTAACTTTCTTTTTTTTGCAATTAGATGGATGAAGTGCTGGCTTCAGTGGCAGAGACTATCAAGAACTTTGCTGTGATATACCTTGTGGACATAACAGAGGTGCCCGACTTCAACACAATGTACGAGCTGTACGACCCCTCAACCGTGATGTTCTTCTTTAGGAACAAGCACATTATGATTGATCTTGGAACTGGAAACAACAACAAGATCAACTGGGCTCTAAAGGACAAGCAGGAGTTTATTGACATTGTTGAGACGGTCTACCGTGGGGCCCGCAAGGGTCGAGGTCTGGTGATTGCACCTAAAGACTACTCCACTAAGTATCGCTACTGA

>*NDUFA13*

ATGACGGAATCGATAATTAGGAACAAGCCAGGAATGGCGTCTGTCAAGGACATGCCCCTCCTCCAAGATGGGCCACCTCCGGGTGGGTTTGCTCCGGTCCGATACGCCCGTCGAATCCCAACCTCCGGTCCCAGTGCCATGGCCATCTTCTTGGCAACGTTCGGTGCTTTCTCTTGGGGCATGTACCAGGTCGGCGTAGGGAACAAGAAGCGCAGGTAACATTAAGATTTCGATACGTAGATTTCTCTTTTGGCAATCTTCGCTGCATTGGTTTCATTGTTCTTTCGCTCGATTGGCTTGTTATTGAACCTGCTCTGTGTCCCTTCTGCTAATTTCAATTTGGGGATCTTCGGTGATTGTTCGAATTGCGAATGCCCTAGAGATATATGGGTTTTTTTTTAATCAGAGGCGTTTGTTGGGAAATTGGAAGAATTTTCTGACTGATTTTCTCGTGTACCTAGGTTAGATTTTGATCTGTTGATTATGCTGAAACGTAGGCTTAAGAAGTGAATAAAACATACATTTGAGGCTCCGTGGCTAATTGCTTGGCAGTTGAATCATTAACTGCGGTCATAAAAGATCATACTGAAATTAGGATTCTGTAGAGGTTGGGAAATTTGGATTTGTTGTTTGCTGATTTTTTGGAAGGGGGATACATTGCATAGTCAATAATAGAAGAGGCTGGGAATTTTGGATTTGTTGTTTGCTGATTTTTTTGTAAAGGGGATAAATTGCATAGTCAATTATACATGTTTGCTGCCTGCTCCTTGAAAATGTGAACTGAGATTGTTAAAAAAACTTTGTTCTTAAACTACATAGCTTTTTCATTTTTGTTCAATGAAGCTATGTGACTATGAGTCAGTTAACATTGATTTCTGGCATAGTAGAATTGCATTTGATGATTTTAAATGGGCTGAATAAGGTCTTTCATGTCTTCTATGTTGAGTAATTCATTTAGAAGATTTGAGGACTTTCAGCTGTTGAGATATGGCCCTTATCATAAGGGTCATAGACTCATAGTCATTGTGTTAAGCATCATGAGTCATGATCTTTGCCTTTGAAGTATCTGTTAGACAGTCCATTTTCAGGGATGCTGAAGTTGAAATCCTTGGCATTGCTGGATTTATCAATTAAAGGCCGCTGGAAATTATATGTCTCAGAAATTTCTCAACTAGAATAGTTGCTTTAAATACAGACTCTTGCAACAAAGAATTGGATTTTGGGAGAATATCAAACTAATTACAATAATTTCCCTTTGATGATGTTTGTTTTCTAAGACAAATCATTATAATTTATAAATGTTTAACTATTACTGTGTGGTGACCAAGGCATATATGTGGAGTTCGCTGGTTATATTCATTTTGCAATTCACTATTTCTTATCTTTATCCATTGGTAATGCATTTGAAATTAGGTAGCTAGTTAGCCAAGTAACGTTAGTCTGTTCATTTACAATTATTAACTGAACAGATGAGATGAATTTACATTGTTCTATACGTGTATAGTTTTTTCAGACTTGTGAAGTAGAATGAAACTAGTTTGAGATGTTGATAAATTCCTATGAATTGTCACTTTATAATAAAACTGCGTGTACAGGGCACTTAAGGAGGAGAAATATGCTGCTAGGAGAGCTATACTACCTTTTCTGCAGGCAGAAGAGGATGAAAGGTATCTTACTATTTCATTACCCTGGAATCATCAAACACTGGCTATGGTGATCAATCTGTGAGATACGTAATTTTCTGTATTTGCTATACCTTCGTGCAGGTTTGTGAAAGAGTGGAAAAAGTATTTGGAAGAGGAAGCCAGAATTATGAAGGATGTACCTGGTTGGAAAGTTGATGAGAGTGTTTACCACTCTGGTAGATGGATGCCTCCTGCCACAGGTGAACTTCGTCCTGAAGTCTGGTAA

>*PcMYB4*

ATGGGGAGATCGCCATGTTGCGAGAAGGAGCACACCAACAAAGGAGCATGGACGAAAGAAGAAGACGACCGTCTTGTTAACTACATCAAATCTCATGGAGAAGGCTGTTGGCGTTCGCTCCCCAAAGCTGCTGGTTCGTTTTTTCTTTCTCACAACCCCTAAAAAACATTCTTCATTAATTCTTTCTTCAATCACCAGAAATTTCTGGAAACAGTTTTTCTTCATCTTCGTCTTCGTCTTCGTCCTGCCCCCAAAAAAAAAGAGGAAAAAAATTCCATATATAACAAGTTATGGTCTCTAGGCATGCTTTTGTATTATAGTGGTCCAAGTTTTAATGCGTACCTAAAAGTTACAACAGCCGAGCAAATTAAGAATATATAACAACAATTGATTTTGTGTGTTTAGATGTTTACATGATTCAAAAAACAACAAATGATCGATTTGGTGGCATGGGTAAATTGAATTTAGAACAGTCGATCTTCTTATGGGGTTTGATCGGTTTGTGTTGTTTGTACGTGTATTACTAAGTCTACTAACTATTCTTGTTGTTGGGTAGGTGGAGTTGTATTTATGACATTTTTTTTGTATACCCATCTGATTAAATTTTAATTGCCTTGCAGGTCTTCAAAGATGCGGAAAGAGCTGCAGATTGAGATGGATAAACTACCTCAGACCTGATCTCAAGAGAGGAAATTTCACCGAGGAAGAAGATGATCTCATCATCAACCTTCACAGTTTACTAGGCAACAAGTAATTTCAAAGACCCGATATATATATAGTTCTTGTGTTCCAATTTTCATGCGTGTCTTATTCTCGATCGAGAATTAAATTGACGTGTTGCATTATTGTTCTTGTTTTCTTGTAGATGGTCTCTTATAGCGGCTCGTCTTCCAGGACGAACCGATAATGAAATTAAGAATTACTGGAATACCCACATCAAAAGAAAGCTTATTACGCGCGGGATCGATCCTCAAACTCACCGTCCTTTTCATGCTTCTTCATCTCCAAACAACAAGAATGTTAGCAATGTAACGACCATGGCCACTTCAATTGATACCGCTACAACCACACAAACACAAAATGGAGGGTTCCAATTGATCAACATACAAAAAGGCTCTCTCTTGCAATTCGCTCCCACGCCCGAAACAAAAAACCCTAGTTTCACAAGACATGTGGGAAATTGTGCTCCCATTGTCGGAATCTCGGGCCGTGATGAAGATTCTAACAATAGCGTTGTGACGGGTGAAGATCAATTACTCGAAGGAGTCAACCTCGAGCTCTCTATTAGTCTTCCTTCTCCTCCGGCGAGGGTATCGCCTAGCAATGTCAAGCAAGAACAACAACAACAACAACAATATTCGTACTTGTGGAGAGGTTCAACCAACACATCAAGGCAATTGCCGGTGGCTCAACAAGGAGTTTGTTTGTGTTGCCATCTAGGGTTTCAAAACTCCAAATCTTCGTGTAATTGTTTGTCAATGACAACAACAATCACATATGGCCAATGA

>*PcPAL*

ATGGAGGTTTCAAACGGGCATTGCAATGGAAACGGCGTCGCTTTGAACGGGCTATGCTTGAAGGAGGCGGCGAAGGTGAAGGCGTTAGCGGACCCGTTGAACTGGGGAGAGGCGGCCGAGGGGATGAAGGGGAGCCATTTGGACGACGTGAAGAGGATGGTGGAGGAGTTTAGGAAGCCGGTGGTGAAGTTGGGGGGAGAGACGCTGACGGTGGCGCAGGTGGCTGCGATTGCGGCGGCGGAAGAGGGTGGAGTGACGGTGGAGCTAGCGGAGGAGTCAAGGGCGGGGGTGAAGGCGAGTAGTGATTGGGTGATGGATAGCATGGACAAAGGGACGGATAGTTATGGGGTTACTACTGGATTTGGAGCCACGTCTCACCGCAGGACTAAGAACGGTGGTGCTCTCCAAAAGGAGCTTATTAGGTGATGATAATTCAATAGTTGACCCTTTTTTTCTATTTTTCATAAAAATGATATTCTTTTTTTTGTTTGATCTTCGGTGATATGAGATGCTTCTTTTGTATATGCATGATTATGGTGAGGTATGATTCTGGTGAAATTTATGTGCTAAAAGTTAATTGTAGGGAAATACTTAAGTTGATCATCTACCTTGGCAATGTTTTTACTTATGATTATAATTAATTATAGTTTAGGTTGTGTACAACATTAAATAAGTTGGAAAAAAAAGTTGAAATCATATACCGATACAATTTTTTCTCAATTTAATCCGACATATATGAGATTATATGAAATAAATACATGATTTTTTTATTGAGGAAAAACAAACTAACCAAGTTTTTTTTGTAAGAAGAATTTGATTGTGTGATTGTCATTAAATTGAAGGTTGGTTAGATAGTGATGTTGTTGGTAGGCTTTGACTTGGTCCTTGCGGCTTGATTCAGGGGTAGGTAGGGGCCGGCATTCTAATTTCTAAAATTCACCTACCAACCAAACCGTAAATTTCAATACTCCTTATAATTCTTTCCCTTTAAGCTTTACTTACTAAACTTTCTGCCTAATGGGCCCACAGGCTGTCTTAATTAATTAGAAGTTAATCATTATGATTACTTATTTAATACGGAGTAGTAATGAATAAAGGAAAAGATTGTGACAAGTGTGTGTAGAAAGTTAGGTGTTTTCTTTTGATGACACTTCGATTGATTAATATTTAGAGTATCTTATAAAATGGTTAATAAGTAAATTTCTTCAAAATGTTTTGTAGGGGTTCTAATTAAGGCTCATCCTAAAGATAAATCCCTCAATTCCTTTAATATTACGTATCATAACTAATATGCATAGTAACATTTATTTTAAAATAAAAATGTTATTTCCTTTAAAAAGTATACCTGCAATAGATTTCCATCTTGCAAATCCAATTAATTAAGAAAAAACTCGAACCTTTTGTTTCTATGTAAAAATTATAGTGCCCATCTACTGAAAGATCAACCTAATCATCCTTTCTTATAATATACAAAATATTTGAATTGAGAAAGAACACCTTTCAAGTTATGAAGTAAAAGCAACTTTAATTTATGAAGTAAAAGCAACTTTAATTCTATGTGTTTTGATATATATAAAGAGATTGATGTATCAATTATTTTAGAAGACTAAATGTTAAGTTTGAACTGTCTCTATCATATGATCTTTGTTTATTTTTGAGTAATCTATCATATGATCTGATTAGTCATTGAATTATGCTGCATCTTAATTTTTATTTATTTATTGTCAAGCATTATGAACGTTAATTAGGAAAATTCTGAAATTGAAAATTGACACGCAGATTTCTGAACGCGGGAGTTTTCGGCAACGGGGTGGAATCATGCCACACCCTCCCCCACTCCACCACCAGGGCCGCCATGCTTGTACGGATCAACACCCTCCTCCAGGGCTACTCCGGCATCCGCTTCGAGATCCTCGAAACCCTCGCCAAGTTCCTCAACACCAACATCACCCCCTGCCTCCCTCTCCGCGGCACCATCACCGCTTCCGGTGACCTCGTCCCCCTCTCCTACATCGCCGGCCTCATCACCGGCCGGCCCAACTCGGTCGCCGTTGGCCCCGACGGCCGCCGCCTCTCCGCCTCCGAGGCATTCCAGCTCGCCGGCATCGAATCCGGCTTCTTTGAACTCCAGCCGAAGGAAGGCCTCGCCATGGTCAACGGCACCGCTGTCGGCTCCGGCCTCGCCTCCATGGTCCTCTTCGACGCCAACATCCTCGCGGTGTTCTCCGAGGTCCTCTCCGCTCTCTTCGCCGAGGTCATGAACGGAAAGCCAGAGTTCACCGACCACCTCACGCACAAGCTGAAGCATCACCCCGGCCAGATCGAGGCCGCTGCTATAATGGAGCACATTTTGGACGGATCTGGCTACGTGAAGCACGCGGAGAAGCTCCACGAATTGGACCCTCTCCAGAAGCCCAAACAGGACCGATACGCCCTTCGTACTTCTCCGCAGTGGCTCGGCCCTCAAATCGAAGTGATCCGAGCGGCTACCAAGATGATTGAGAGAGAAATCAACTCCGTCAACGACAATCCGTTGATCGATGTTTCCAGAAACAAGGCTCTGCACGGCGGAAACTTCCAGGGAACTCCGATTGGAGTTTCCATGGACAACACCAGGCTTGCCCTAGCCGCGATTGGAAAGCTGATGTTCGCTCAATTCTCCGAGCTCGTGAACGATTTCTACAACAACGGATTGCCGTCGAATCTCTCCGGCGGGAGAAACCCTAGCTTGGACTACGGCTTCAAGGGAGCGGAAATCGCGATGGCGTCTTACTGCTCAGAGCTGCAATTTCTAGCTAATCCGGTGACGAATCACGTCCAGAGCGCGGAGCAGCACAACCAGGACGTCAATTCCCTGGGCCTGATCTCGTCAAGAAAGACCGCTGAGGCGGTGGAGATTCTCCAGCTCATGTCCTCCACGTTCCTGGTGGCTCTGTGCCAAGCGATTGACCTCAGGCATCTGGAGGAGATCCTCAGGAACACCGTCAAGAACACAATCAGCCAAGTAGTGAAGCGAATATTGTCTGTCGGCGTCAACGGTGAGCTCCATCCGAGCAGATTCTGCGAGAAGGATCTTCTCAGAGTAGTCGACCGCGAGCACGTGTTCGCCTACATCGACGATCCTTGCAGCCCTAACTACGTCCTGATGCAGAACCTCAGACAAGTTCTGGTGGATCACGCCCTAGAAAACGGCGACAAGGAGAAGACCACCGCAACTTCGATCTTCCAGAAGATCGGTGCGTTCGAGGAGGAATTGAAGGTTGTGTTGCCAAAGGAGGTTGAAGGAGCAAGAAACGAGTATGAGAATGGAACTTCTGGAGTTGAGAACAGGATCAAGGAATGCAGATCGTTCCCGTTGTACAAGTTCGTGAGGGAGGAGCTCGGGACGAGCTTGTTGACTGGGGAGAAGGTTAGATCGCCTGGGGAGGATTTCGATAAGGTGTTCACTGCGATTTCTCGAGGATTGATGATTGATTCCTTGTTGGAATGCTTGAAGGAGTGGGATGGTGCTCCTCTGCCTATTTGCTAG

>*PcSTS*

ATGGCAGCTTCAACTGAAGAGATGATGAAGGCACAAACAGCCGCCACCGTCCTGGCCATCGGCACGGCCAATCCTCCCAATTGCTACTACCAAGCTGACTTTCCCGACTTCTACTTCCGTGCCACCAACAGCGACCACCTCACCCACCTCAAGCACAAATTCAAGCGCATTTGTATGCCTCCTTATATATGCTTTCACTTATATACTATGCATACAATTCCTCTACTCGGGTTTGTTTGATATATGTACCCAATAAAATAAGCTAAGATAATTAATCGATATGCATAACAACAACATAGATGCTTGATAAATATTGAAATTCCATTTCCTTGTTTTTTTTCTGGGGTTTTGTTTGTATACACCCACCCGTACGTAGGTATCTCGATCAATTCGCACTTCCGATACGTGCGCATCCGAAATGCCGGATGATCAATATATTAATATTCTTTTGTATTAATGTACGATTCTATGAGTCTTACAAAGTTCTTTAATTTTTTTTTTTGGTACGCCTCCTAGAATATTTAATTTCATTTTTTCGAACGCTTTTAAGATGCTTACATTATGTGTGGGCTGGTTTTGAAATTTTTTATGAGTGTATTTAAAGAGCGTGTATTTTTTCATGATAATAGGTGAGAAGTCAATGATCGAGAAGCGTTACCTTCAATTGACGGAAGACATTCTCAAAGAAAACCCGAATATCGGTGCGTACGAGGCACCATCATTGGATGTAAGACACGAAATTCAAGTGAAAGGAGTTGCACAGCTTGGGAAAGAGGCCGCTCTCAAGGCCATGCAAGAGTGGGGCCAACCCAAATCTAAGATCACACATCTCATCGTGTGTTGCATAGCCGGGGTTGACATGCCAGGCGCAGATTATCAACTCACTAAGCTTCTTGACCTAAACTCTTCTGTTAAGCGCTTCATGTTTTACCACCTAGGTAAGTATAAGGGGTCATCCAATGACTTATAGAGACCTTGTTTAAATTATTAAATTGAGGCCCTAATCTACGTACATATATAGGATGTTACGCTGGTGGCACCGTCCTTCGTCTTGCCAAGGATATAGCCGAGAACAACAAAGGAGCTCGTGTTCTCATCGTTTGTTCAGAGATGACGCCAATCTGCTTCCGTGGGCCATCTGAAACCCATATAGACTCCATGGTAGGGCAAGCAATATTTGGTGATGGTGCTGCAGCTGTCATAGTTGGAGCGAACCCAGACCTAACAGTTGAGAAGCCCATTTTCGAGTTGATTTCCACAGCCCAAACTATCATACCTGAATCTGATGGTGCGATTGAGGGCCATTTGCTAGAAGTTGGACTCAGTTTCCAACTCTACCAGAATGTCCCCGCACTAATCTCTAATAGCATAGGAACATGCCTTTCAGAAGCTTTCACCCCTCTAAACATTAGCAATTGGAACTCCCTCTTCTGGATCGCACATCCTGGTGAGTATATTGTATATGATTTATGGGTTTTTTCTAGTGCGTGAATAATCTAATGTTGCAAGTAATATCGATGCGATTCCGCAAATTAGAATGGATTAACCTTGAACAAATAATTTTAAAATTAATAAGCATTAATTAAGTTTAATATACTTCATAAACGTGAAATTATATATGTGTCCAAGATATATAGGTCCGGTGGCTTTCAGTTTGGCTCATTGTATATATATGTCCAAAAGTTCTTTATTCTTTTTTGCCCAAAAAAATTCTTTTTTCAAGTTGTCAGAATGATAGTTGTGTGGATTTTACAATTCTGATTACGGGTCACTCAGATTTTTTGGTTTTTTCGAATATAAGTTAATTTAAGATCATGTATAACTTATATATTAAATAATGTTGATTTTTATTCCTATTTTTGAGGACATTATCATATATATATATATAACTAATTTAAAATACGATATCAATCTATCTCATTGTACAGGTGGCCCTGCTATCCTAGACCATGTTGAGGCCACCGTTGGTCTCAACAAGGAGAAACTTAAGGCAACCAGACAAGTGCTGAACGACTATGGAAACATGTCAAGTGCTTGTGTGTTTTTTATCATGGATGAGATGAGGAAGAAGTCACTTGAAAACGGCCACGCAACGACTGGAGAAGGACTGCAGTGGGGCGTTCTGTTTGGATTCGGGCCTGGTATTACTGTTGAAACTGTGGTGCTACGAAGTGTGCCCATCATTTAA
